# Polymer-Bound and Macromolecular Hindered Phenolic Antioxidants: Architectures, Anti-Migration Mechanisms, and Applications

**DOI:** 10.3390/polym18202601

**Published:** 2026-10-21

**Authors:** Yukun Jian, Jiajun Xu

**Affiliations:** 1 College of Chemical Engineering, Zhejiang University of Technology, Hangzhou 310032, China; 2 School of Chemical Engineering, Ningbo Polytechnic University, Ningbo 315800, China; 2025013@nbpu.edu.cn

**Keywords:** hindered phenol, macromolecular antioxidant, anti-migration, polymer-bound stabilizer, hyperbranched polymer, polyolefin stabilization, polymer recycling

## Abstract

Hindered phenolic antioxidants are the primary defense against thermo-oxidative degradation in most commercial polymers, yet in many long-lived service environments, their effectiveness is limited less by radical-scavenging chemistry than by physical loss—migration into contact media, extraction by service fluids, and volatilization during processing. This review examines polymer-bound and macromolecular hindered phenols, in which the phenolic function is grafted onto preformed polymers, incorporated by copolymerization, multiplied in dendritic and hyperbranched architectures, or carried by hybrid and bio-based scaffolds. Each architecture family is analyzed against common criteria—structural control, phenolic hydroxyl content and retained activity, compatibility, and scalability—with study-level quantitative analysis of representative studies, selected for the completeness of their quantitative retention and performance data. Anti-migration mechanisms and the methods used to verify retention are critically assessed; in particular, survival of antioxidant activity after extraction is treated as evidence of functional retention, not as proof of covalent attachment, which requires independent structural characterization. Application performance is then reviewed across packaging, infrastructure, electrical insulation, elastomers, engineering plastics, biomedical polyethylene, and recycling. The strongest unmet needs are methodological: standardized extraction-based verification of covalent binding, quantification of phenol utilization efficiency in solid hosts, and long-term validation under sector-relevant protocols.

## 1. Introduction

Most commercial polymers owe their useful lifetimes to stabilizers rather than to intrinsic stability. Under the combined action of heat, oxygen, light, and mechanical stress, hydrocarbon-based polymers undergo autoxidation, a self-accelerating radical chain reaction. Initiation—by heat, light, or trace-metal catalysis—first generates primary radicals, and these radicals then abstract hydrogen from the polymer backbone; the resulting carbon-centered radical adds oxygen within milliseconds to form a peroxy radical; and the peroxy radical abstracts hydrogen from a neighboring chain to form a hydroperoxide while regenerating a carbon-centered radical [1,2]. The kinetic danger of this cycle lies in its branching step. Hydroperoxides decompose thermally, photolytically, or under trace-metal catalysis into alkoxy and hydroxyl radicals, each of which starts new chains, so oxidation accelerates autocatalytically once a critical hydroperoxide concentration accumulates. The macroscopic consequences, namely embrittlement, discoloration, cracking, and loss of mechanical and dielectric properties, follow directly from the molecular events: chain scission lowers molar mass, oxidation introduces polar chromophores, and crosslinking destroys elasticity.

**Figure 1:**
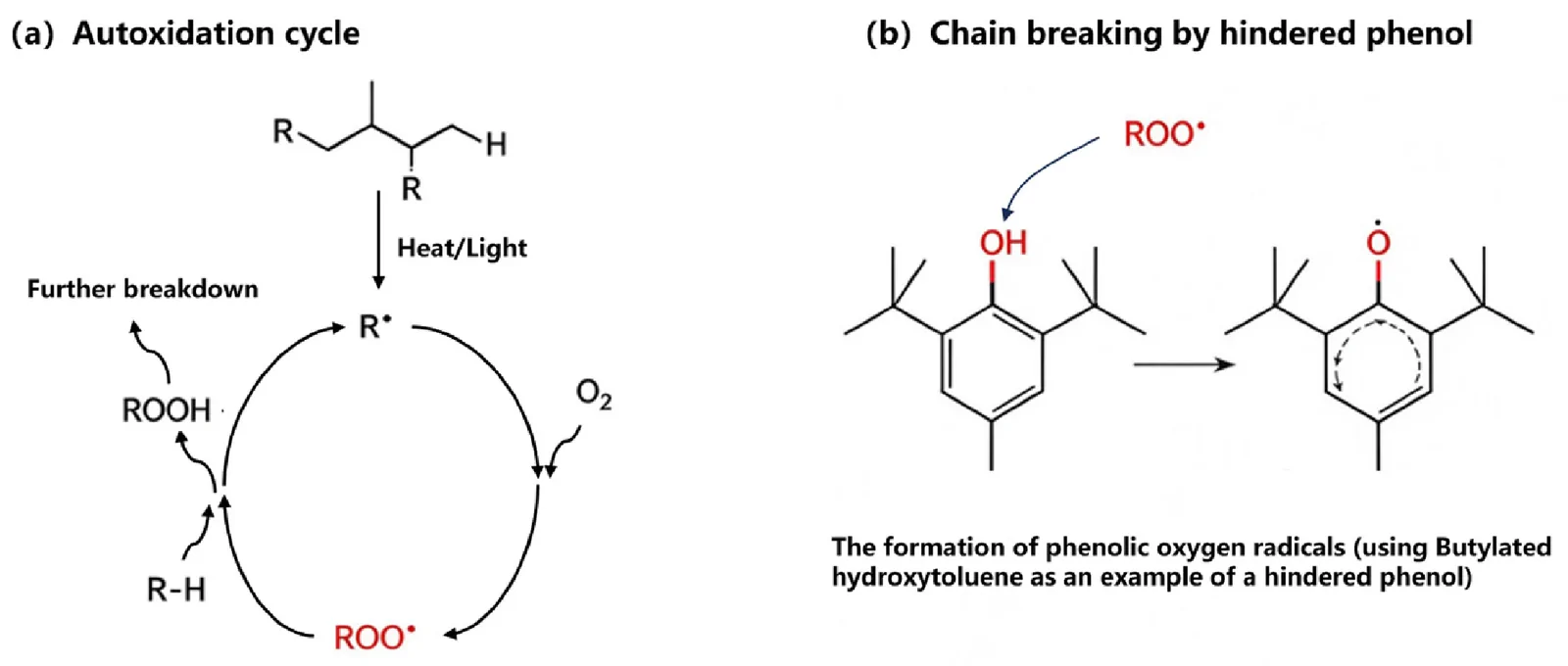
(**a**) The autoxidation cycle of hydrocarbon polymers: an initiation event generates the first radicals, propagation carries the chain through carbon- and oxygen-centered radicals, and hydroperoxide decomposition branches it autocatalytically. (**b**) Chain breaking by a hindered phenol: hydrogen transfer from the phenolic OH group to a peroxy radical gives a resonance-stabilized phenoxyl radical (shown for butylated hydroxytoluene, BHT) that is too unreactive toward the polymer substrate to propagate the radical chain. The direction of the arrow indicates the sequence in which the reactions occur.

Stabilization strategies follow directly from this mechanism. Chain-breaking (primary) antioxidants intercept the propagating radicals, while hydroperoxide-decomposing (secondary) antioxidants such as phosphites and thioethers remove the branching agent before it can spawn new chains [2,3]. Hindered phenolic antioxidants are the most widely used class of primary antioxidants in polymer stabilization. Acting as chain-breaking donors, they transfer their phenolic hydrogen to peroxy radicals and thereby interrupt the autoxidation cycle. The resulting phenoxyl radical is resonance-stabilized by conjugation of the oxygen lone pair with the aromatic π-system and is further shielded by its flanking tert-butyl groups; it is therefore far less prone than a peroxy radical to abstract hydrogen from the polymer substrate, so chain propagation is interrupted rather than transferred (Figure 1). This stabilization is not merely a theoretical construct: the phenoxyl radical derived from 2,4,6-tri-tert-butylphenol is persistent enough to be observed directly in solution by electron paramagnetic resonance spectroscopy, and the related galvinoxyl radical is a commercially available, shelf-stable free radical routinely used as a calibration standard. The phenoxyl radical is then consumed by several competing pathways—coupling, disproportionation (which partly regenerates the phenol), reaction with further peroxy radicals to give peroxycyclohexadienones, and quinone formation—rather than by a single elementary termination event; the quinoid products formed along these pathways are the chemical origin of phenol-related discoloration [1,2,4]. This chemistry is mature. Structure–activity relationships connecting O–H bond dissociation enthalpy, steric environment, and substitution pattern to antioxidant efficiency have been established over decades, and the transformation products of hindered phenols, including quinones, stilbenequinones, and coupling products, are well characterized, not least because they govern discoloration in phenol-stabilized polyolefins [2,3].

What is far less mature is the control of where the antioxidant molecule is, and for how long. It has long been recognized that the lifetime of a stabilizer in a finished polymer article is frequently determined not by chemical consumption but by physical loss—diffusion to the surface followed by migration into a contacting phase, extraction by service fluids, or volatilization [5,6]. The transport physics of this loss is well described by Fickian diffusion through the semi-crystalline matrix combined with interfacial partitioning into the contacting medium [7,8,9], and its practical consequences recur across application sectors: in food packaging, where phenolic antioxidants and their transformation products are detected migrating into simulants from essentially all commercial polyolefin articles examined in the surveys reported to date [10,11,12,13,14,15]; in pipes and geosynthetics, where depletion against decades-long design lives is the limiting factor [16,17,18]; and during melt processing itself, where volatility and dispersion already erode the stabilizer inventory [19,20].

The generic response to physical loss is to increase the effective molecular size of the phenolic species, and ultimately to bind the phenol covalently to a polymer chain. This idea now has a literature spanning several distinct molecular architectures and more than two decades. Reactive-processing routes graft polymerizable hindered phenols onto preformed polyolefins in the extruder [21,22,23,24,25,26,27]. Copolymerization and step-growth routes build the phenol directly into the chain during polymerization [28,29,30,31,32,33,34]. Dendritic and hyperbranched syntheses maximize the number of phenolic groups per molecule [35,36,37,38,39,40,41,42]. Dedicated macromolecular phenols have been developed for the demanding environment of elastomers [43,44,45], and hybrid or bio-based scaffolds, including polyhedral oligomeric silsesquioxane (POSS) cages, graphene oxide, nanosilica, and lignin, provide high-molar-mass carriers with added functionality [46,47,48,49,50,51,52,53]. The wider landscape has been recently reviewed from a general perspective, covering both general strategies for integrating antioxidant functionality into polymer materials [54] and anti-migration antioxidants for polypropylene [20]. Neither, however, is restricted to hindered phenols, and neither attempts a cross-architecture comparison under unified evaluation criteria, which is the specific gap the present review addresses.

Several forces make a dedicated, critical review of this field timely. Regulatory scrutiny of migrating substances in food-contact materials continues to increase; infrastructure applications demand validated 50–100-year stabilization; the clinical success of vitamin E-stabilized ultra-high-molecular-weight polyethylene (UHMWPE) demonstrates that retention-first stabilizer design can succeed in a regulated, high-value market, although that clinical success also rests on crosslinking, residual-radical control, and processing history rather than on retention alone [55,56]; and the circular economy has made restabilization of recyclates (through multiple heat histories that erode the stabilizer inventory) a routine industrial operation in which low-loss antioxidants hold a natural advantage [57,58,59,60,61,62,63,64,65]. Yet the macromolecular-phenol literature remains fragmented: architectures are rarely compared under common conditions, retention claims rest on heterogeneous tests, and quantitative design rules are scarce.

This review therefore has three aims. The first is to define the physical-loss problem quantitatively, using the packaging, pipe, and geosynthetic evidence base (Section 2). The second is to establish how anti-migration performance and stabilization efficiency are actually measured, including the mechanisms, the methods, and their caveats, so that reported performance claims can be read critically (Section 3). The third is to compare the molecular architectures of polymer-bound and macromolecular hindered phenols under a common set of evaluation criteria, with study-level analysis of representative quantitative work in each family (Section 4), and to assess how these materials perform across application sectors from packaging and infrastructure to biomedical devices and recycling (Section 5), before identifying the methodological and scientific gaps that should orient future research (Section 6).

Relative to these existing surveys, the present review differs in three respects. Compared with the general survey of strategies for integrating antioxidant functionality into polymers [54], we restrict attention to hindered phenols and to macromolecular or polymer-bound implementations, which permits study-level quantitative comparison instead of broad coverage. Compared with the polypropylene-focused anti-migration review [20], we cover all polymer hosts and organize the field by molecular architecture under unified evaluation criteria (structural control, phenol content and retained activity, compatibility and dispersion, and scalability). Compared with the review centered on dendritic and hyperbranched additives [42], that family is treated here as one of four architectures assessed on equal footing. To our knowledge, no previous review has combined an architecture-based classification of hindered-phenol stabilizers with extraction-referenced retention data and a sector-level application analysis.

Literature identification and selection. This article is a critical narrative review rather than a systematic review; nevertheless, its literature basis is described here transparently. Studies were identified by searching the Web of Science, Scopus, and Google Scholar databases (searched up to August 2026) using combinations of the terms ‘macromolecular antioxidant’, ‘polymer-bound antioxidant’, ‘hindered phenol’, ‘anti-migration’, ‘non-migrating antioxidant’, ‘grafted antioxidant’, and ‘reactive processing’, complemented by backward and forward citation chasing from the surveys cited above [20,42,54]. English-language peer-reviewed journal articles were included when they (i) reported a structurally defined macromolecular or polymer-bound hindered phenol, or a comparator system directly relevant to the retention question, and (ii) contained quantitative retention or performance data; conference contributions, patents, and industrial literature were excluded except where explicitly identified as industrial context. Within each architecture family, the studies selected for in-depth discussion were chosen for methodological completeness—specifically, for pairing a retention measurement with a performance measurement—rather than for the magnitude of the reported effect.

## 2. The Physical-Loss Problem: Migration, Extraction, and Volatilization of Conventional Hindered Phenols

**Figure 2:**
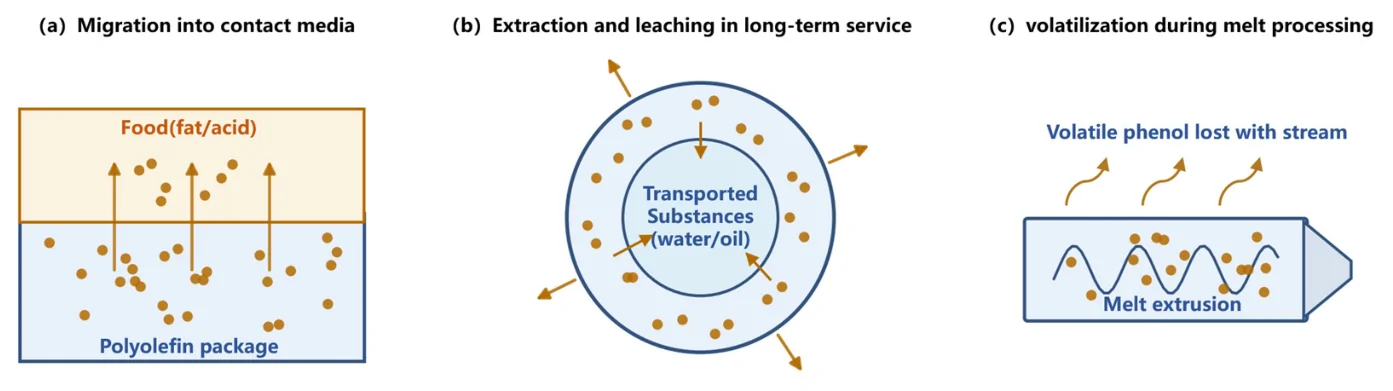
The three physical-loss channels of conventional low-molar-mass hindered phenols: (**a**) migration into contact media; (**b**) extraction and leaching in long-term service; (**c**) volatilization during melt processing. The direction of the arrow indicates the direction of the antioxidant migration.

As outlined in the Introduction, a stabilizer is depleted by two parallel routes—sacrificial chemical consumption while trapping peroxy radicals [1,2] and physical loss by migration, extraction, and volatilization [5,6]. These two routes are conceptually distinct but mechanistically coupled: a phenol that has migrated toward an oxygen-rich surface layer is consumed faster, and the mobility of its transformation products may differ from that of the parent molecule. This section examines the second route quantitatively; the underlying transport physics (Fickian diffusion through the semi-crystalline bulk combined with interfacial partitioning [7,8,9]) is taken up in Section 3. The evidence is organized by loss channel—migration into contact media (Section 2.1), extraction and leaching in service (Section 2.2), and volatilization during processing (Section 2.3)—and the three channels are summarized schematically in Figure 2.

The structural origin of this vulnerability to physical loss lies in the phenol molecule itself. A phenolic hydroxyl group bonded directly to an aromatic ring is neither a purely polar nor a purely nonpolar function: the aromatic ring lends affinity for hydrocarbon media, while the hydroxyl group, although sterically shielded in hindered phenols, retains enough polarity to interact with water, alcohols, and other polar service fluids. Conventional low-molar-mass hindered phenols therefore exhibit appreciable partitioning into water, polar solvents, and nonpolar fatty or hydrocarbon media alike, and the three physical-loss channels examined below map directly onto this solubility profile: migration into fatty and hydrocarbon contact media, extraction by water and chlorinated water, and leaching by polar service fluids such as ethanol or ethylene glycol. Even where the aqueous solubility of a hindered phenol is low in absolute terms, decades of continuous contact with flowing water extract the stabilizer progressively, as the pipe studies discussed in Section 2.2 show. Macromolecular and polymer-bound designs address the problem at this root: by tying the phenol to a large or immobile scaffold, they shift every partitioning equilibrium decisively toward the polymer phase, whatever the contacting medium.

### 2.1. Migration into Contact Media: Evidence from Packaging Studies

The most extensively documented manifestation of physical loss is the migration of phenolic antioxidants from polyolefin packaging into food and food simulants, driven by regulatory interest. A systematic survey of 26 commercial polypropylene food-contact films by ultra-performance liquid chromatography coupled with quadrupole time-of-flight mass spectrometry (UPLC-MS/QTOF) illustrates both the ubiquity and the chemical complexity of the phenomenon [10]: 76 non-volatile migrant compounds were identified across four food simulants, roughly three-quarters of them non-intentionally added substances (NIAS), dominated by the transformation products of Irganox 1010 and Irganox 1076, direct evidence that the additive package present in a commercial article is already a mixture of parent compounds and transformation products before any food contact occurs. Migration of the parent phenols and their transformation products was measurable for essentially all films, and six of the 26 films exceeded a specific migration limit (SML) of EU Regulation 10/2011—five owing to the N,N-bis(2-hydroxyethyl)amine group and one owing to the phenolic antioxidant Irganox 1076, which reached 85 mg kg^−1^ in 95% ethanol against an SML of 6 mg kg^−1^. The important point for the present review is not regulatory compliance but the mere fact of detectable transfer: any molecule that can be measured in the simulant is a molecule no longer protecting the polymer.

**Figure 3:**
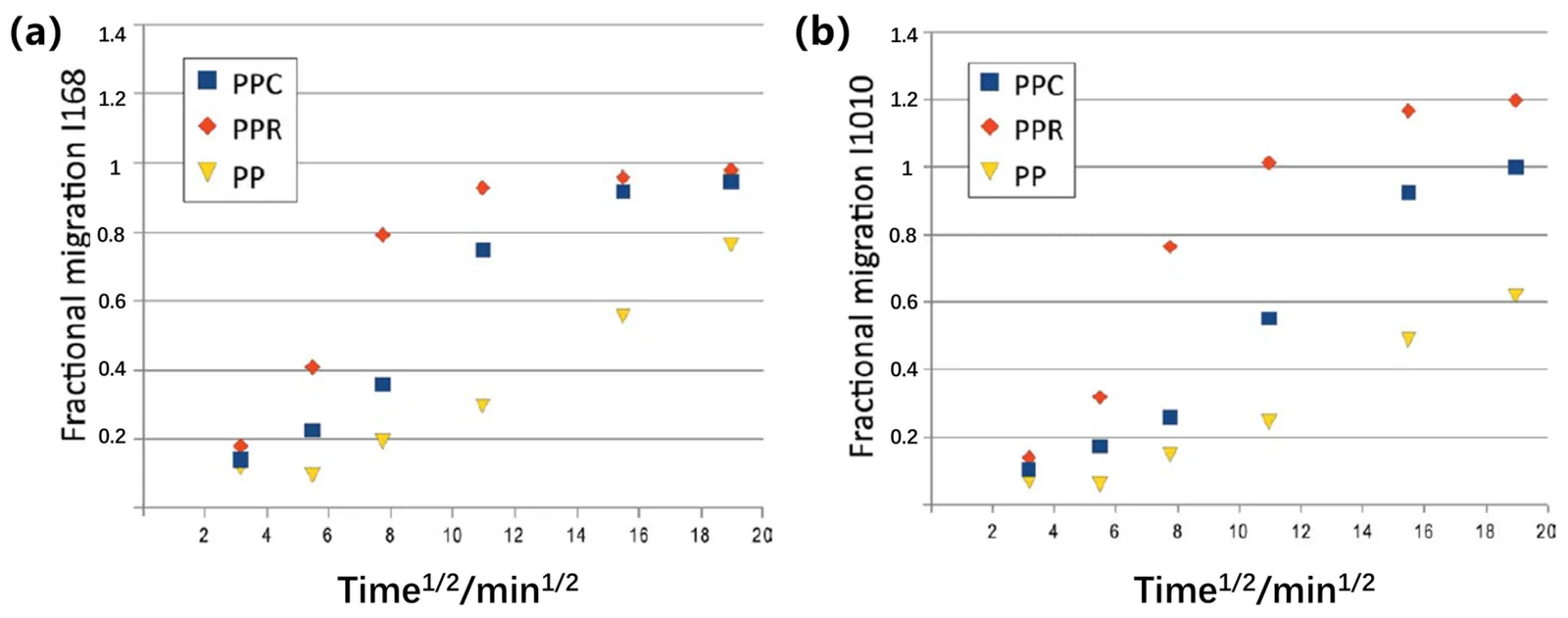
Migration of (**a**) Irgafos 168 and (**b**) Irganox 1010 from three polypropylene grades (homopolymer PP, block copolymer PPC, random copolymer PPR) into 90/10 isooctane/ethanol at 80 °C under microwave heating (note: the *x*-axis is the square root of time, t1/2, in min1/2). Reproduced with permission from ref. [12]. Copyright 2010, John Wiley and Sons.

A second study quantifies how strongly the host matrix modulates this loss [12] (Figure 3). Three polypropylene grades, a homopolymer, a propylene–ethylene random copolymer, and a propylene–ethylene block copolymer, were compared during microwave heating in contact with food simulants. The migration rate of Irganox 1010 and Irgafos 168 decreased with increasing crystallinity (41% for the homopolymer versus 33% for the block and 29% for the random copolymer), and the homopolymer was the most migration-resistant, particularly against fatty simulants. Because the three grades also differ in comonomer distribution, morphology, and amorphous-phase characteristics, crystallinity is best read here as the leading rather than the sole causal variable. At 80 °C in isooctane/ethanol, the migrated amount of Irganox 1010 after 30 min was 535 µg dm^−2^ from the random copolymer against 77 µg dm^−2^ from the homopolymer, a sevenfold difference as reported (the original study does not provide replicate statistics), and the corresponding diffusion coefficients differed by a factor of five (1 × 10^−7^ versus 2 × 10^−8^ cm^2^ s^−1^). In 96% ethanol the same ordering held at much lower absolute levels (2 × 10^−10^ versus 3 × 10^−12^ cm^2^ s^−1^), and the smaller phosphite Irgafos 168 migrated at roughly twice the rate of Irganox 1010 throughout. This size ordering is consistent with transport retardation by molar mass, although differences in polarity, solubility, degradation, and partitioning between the two additives may also contribute. The choice of simulant proved equally important. Isooctane, a strongly swelling fatty simulant, caused rapid depletion of both antioxidants and a marked increase in the calculated diffusion coefficients relative to 96% ethanol, while migration into aqueous and acidic simulants stayed below the detection limits regardless of microwave time and temperature. Two design-relevant conclusions follow. First, migration resistance can be improved modestly by matrix engineering through higher crystallinity, but only within the narrow window that processing and mechanical requirements allow. Second, the worst-case combination, a low-crystallinity polyolefin in contact with a swelling lipid phase, is precisely the one encountered in fatty-food packaging and in many lubricant and fuel-contact applications, and it cannot be fixed by matrix engineering alone.

Migration is not restricted to synthetic phenols. The natural phenolic antioxidant carvacrol, incorporated into polypropylene films for active packaging, migrates readily enough that film composition, structure, and function must be characterized together to balance controlled release against retention [15]. In polypropylene-based active films loaded with butylated hydroxyanisole (BHA), butylated hydroxytoluene (BHT), and tert-butylhydroquinone (TBHQ), migration analysis had to be combined with antioxidant and mechanical characterization to assess whether the films retained function after contact [13]. A recent survey of synthetic phenolic antioxidants in food packaging confirmed that their occurrence and migration depend systematically on plastic type and storage temperature, underlining that this is a generic, materials-inherent problem rather than a formulation accident [14]. Fatty simulants in particular have long been known to extract Irganox-type phenols aggressively from polypropylene [11].

The consequences of this loss channel are twofold, and both are quantifiable. On the product side, every migrated molecule is protection subtracted from the package: films whose phenol inventory is depleted by contact with a swelling fatty phase lose their induction period long before their design life, so the package itself embrittles or its contents oxidize sooner. On the exposure side, each molecule measured in a simulant represents potential transfer under the prescribed test conditions—an upper-bound estimate of possible exposure rather than a measured ingested dose, because actual dietary intake additionally depends on the real foodstuff, its contact time and temperature, and consumption patterns. The temperature dependence documented above means that this potential transfer is greatest precisely under the conditions consumers actually impose, such as hot filling, microwave reheating, and warm storage [12,14]. Synthetic phenolic antioxidants and their transformation products are accordingly now tracked as emerging contaminants in environmental and food-contact monitoring; such detection does not in itself establish toxicity or regulatory non-compliance, but it subjects the entire product class to intensifying regulatory scrutiny and toxicological evaluation.

### 2.2. Extraction and Leaching in Long-Term Service

Outside packaging, extraction by water and other service fluids is the dominant physical-loss channel for infrastructure applications. For natural-gas polyethylene pipes operating under internal pressure, oxidation-induction-time data measured over long periods have been extrapolated with Arrhenius-type models to predict antioxidant depletion and service lifetimes of decades [16]. Potable-water and chlorinated-water service, discussed below, is a related but distinct environment in which extraction by water and chemical attack by the disinfectant act together, and gas-pipe lifetime predictions are not directly transferable to it. The magnitude of this loss is sobering. A recent review of lifetime-prediction methods for polyethylene pipes stresses that current extrapolation procedures remain limited: accelerated tests deviate from operating conditions, prediction models are largely empirical, and antioxidant depletion and host aging are not cleanly separated in time, so that lifetime estimates based on oxidation-induction measurements alone tend to be unreliable [66]. The loss accelerates dramatically when the transported water is oxidizing. Accelerated aging of polyethylene pipe grades in aqueous chlorine dioxide, a common potable-water disinfectant held at constant concentration, showed that the oxidizing medium attacks the stabilizer system and shortens the oxidation induction period far more rapidly than hot water alone [67]. In polyolefin geosynthetics, where design lifetimes of 50–100 years are specified, antioxidant depletion kinetics distinguish sacrificial stabilization regimes, in which the phenol is simply consumed or washed out, from regenerative schemes designed to extend the induction period [17]. Environmental exposure of polyolefins to water, air, and compost leads to leaching of stabilizers together with their degradation products, so that physical loss and chemical transformation are coupled in practice [18]. The common quantitative feature of these studies is that depletion follows approximately diffusion-controlled kinetics. Loss is fastest in the first service years and from the surface layer, so the oxidation that follows is initiated at the surface and propagates inward, a failure pattern that no replenishment of the bulk can prevent once the surface is exhausted.

The consequence of this channel is application-dependent, but it is starkest in long-lived infrastructure: performance collapse at the end of a finite induction period, and the pipe studies make the causal link explicit in engineering terms. The depletion models extrapolate antioxidant concentration toward a critical threshold and equate its crossing with end of service life [16,66], and direct failure analysis confirms the endpoint: when three commercial polypropylene random copolymer pipes were hydrostatically aged in 110 °C water, the two pipes whose antioxidant package was fully depleted during the test both failed, whereas the pipe that retained a measurable antioxidant reserve did not: depletion, rather than time per se, marked the critical point beyond which oxidative failure accelerated [68]; field failure of pipes additionally depends on stress state, defect population, and slow-crack-growth resistance, so depletion is best read as a necessary, not sufficient, condition for end of service. The same causal chain has been measured directly in polyamide 11, where tracking the actual hindered phenol concentration during thermal aging shows progressive depletion as the life-limiting quantity, with exhaustion marking the onset of rapid oxidation [69]. The most consequential historical example is medical: first-generation sterilized UHMWPE joint implants, in which residual free radicals met an insufficiently protected matrix, underwent oxidative embrittlement and delamination in vivo, contributing to premature implant failure and revision surgery, the clinical and financial cost that the vitamin E stabilization strategy was developed to eliminate [55]. In all these cases the structure of the problem is identical: extraction and consumption drain a finite inventory, and the end of that inventory, not the intrinsic chemistry of the polymer, sets the lifetime. Leaching simultaneously exports the stabilizer and its transformation products into soil and water [18], so long-term service carries an environmental release term alongside the reliability term.

### 2.3. Volatilization and Processing Losses

A third channel operates at the very beginning of the product life: during melt processing, low-molar-mass phenols can volatilize or be lost with vent streams, and the physical state of the additive strongly affects its dispersion, surface blooming, and ultimate performance, as shown for Irganox 1076 in crosslinked polyethylene [19]. Volatility-driven loss is one reason why BHT-type monophenols have progressively been replaced by higher-molar-mass ester phenols, and why further increases in effective molecular size (oligomeric, polymer-bound, and macromolecular phenols) are now an explicit design goal, as summarized in a recent review devoted specifically to anti-migration antioxidants for polypropylene [20].

The consequence of this channel is primarily economic, and it recurs with every heat history. Stabilizer stripped during one processing step is protection that was paid for but never delivered to the product; in mechanical recycling, where each cycle adds a new heat history on top of the chemical consumption of the previous life, the loss is paid again at every loop: recyclates arrive under-stabilized and must be restabilized at every reprocessing step [57,61]. The scale of this recurring cost is illustrated by a controlled reprocessing study on post-consumer recycled polypropylene: over five consecutive extrusion cycles, the unstabilized recyclate showed the classic degradation signature of progressively rising melt flow rate together with falling oxidation induction time and onset temperature, whereas material restabilized with 1.0 wt% of a phenol-rich natural extract maintained a stable melt flow rate and higher oxidation induction values across all five cycles, matching a commercial synthetic phenolic antioxidant run in parallel [61]. For the circular economy this converts physical loss from a one-time efficiency penalty into a structural, repeating cost, and it is the channel where low-volatility macromolecular phenols have their most immediate economic justification.

### 2.4. Consequences for Stabilizer Design

Taken together, the evidence above defines the design brief for the materials reviewed in Section 4. Each loss channel has been paired with its consequences: migration couples protection loss to consumer exposure (Section 2.1), extraction couples it to induction-period collapse and environmental release in long-term service (Section 2.2), and processing loss converts it into a recurring cost at every heat history (Section 2.3). Physical loss can be suppressed in two ways. The first is to increase the molar mass of the phenolic species, which reduces diffusion coefficients and volatility. The second is to anchor the phenol covalently to a polymer chain, ideally one chemically identical or closely related to the host matrix, so that migration requires transport of an entire macromolecule rather than a small guest. These strategies, and the broader concept of integrating antioxidant functionality directly into polymer materials, have been reviewed from a general perspective recently [20,54]. Before the realized architectures are compared, however, two questions must be settled. By what mechanisms do larger or bonded phenols actually suppress transport? And how are anti-migration and stabilization performance measured, and occasionally mis-measured, in practice? These methodological foundations are the subject of Section 3, and the critical comparison of molecular architectures then follows in Section 4. It should nevertheless be kept in mind that immobilization is not universally beneficial. Covalent tethering can restrict the local mobility a phenol needs to reach radical sites, and burial of the phenolic group within a dense carrier can reduce its accessibility, so in regimes where chemical consumption dominates—high temperatures, thick sections, or high radical flux—a poorly accessible immobilized phenol can underperform a mobile monomeric one. Quantitative comparisons illustrating both sides of this balance are discussed in Section 4.2, Section 4.4 and Section 5.5. The relative weight of chemical consumption and physical loss is therefore matrix- and condition-dependent, a balance taken up again in Section 6.

## 3. Anti-Migration Mechanisms and How Performance Is Actually Evaluated

Section 2 closed with a design brief: suppress physical loss by making the phenol larger, or by bonding it to a polymer chain. Before the architectures built on that brief are reviewed in Section 4, this section establishes the framework needed to judge them. It first examines the physical mechanisms by which size and covalent attachment suppress transport (Section 3.1), then surveys how migration and extraction are quantified in practice (Section 3.2) and how stabilization performance itself is assessed (Section 3.3), including the systematic pitfalls of each method, and finally identifies what is still missing before structure can be connected quantitatively to retention and performance (Section 3.4). Throughout the architecture review that follows, these methods and their caveats serve as the interpretive lens: a performance number is only as strong as the protocol that produced it.

### 3.1. Mechanisms of Migration Suppression

The macromolecular architectures reviewed in Section 4 suppress physical loss through three distinguishable mechanisms. The first is transport retardation by size. Diffusion coefficients of additives in polyolefins fall steeply with increasing molar mass, as established by diffusion and migration modeling studies [7,8], so oligomeric and macromolecular phenols migrate orders of magnitude more slowly than monomeric analogs of similar chemistry. The second is covalent anchoring. Once the phenol is bonded to a polymer chain, whether by grafting, copolymerization, or transamidation, its removal requires either bond cleavage or wholesale extraction of the carrier chain, which converts a diffusion problem into a chemical-stability problem [20,54]. The third is interfacial compatibilization. When the carrier chain is chemically identical or similar to the host, the additive is thermodynamically miscible and does not partition preferentially toward the surface or the contacting phase, which addresses the interfacial mass-transfer step that controls release into contact media [9]. Non-covalent variants, such as supramolecular assemblies and nanoparticle-bound phenols, exploit strong specific interactions or steric entrapment instead of covalent bonds, at the price of lower permanence under aggressive extraction [39,41].

It should be stressed that the matrix itself remains part of the mechanism: crystallinity, free volume, and the physical state of the additive govern mobility even for large species, as the polypropylene-grade comparison of Section 2.1 showed quantitatively [12,19]. No architecture eliminates migration; the realistic goal is to shift the characteristic loss time well beyond the design life, as pipe and geosynthetic depletion studies make explicit [16,17].

### 3.2. Methods for Quantifying Migration and Extraction

**Figure 4:**
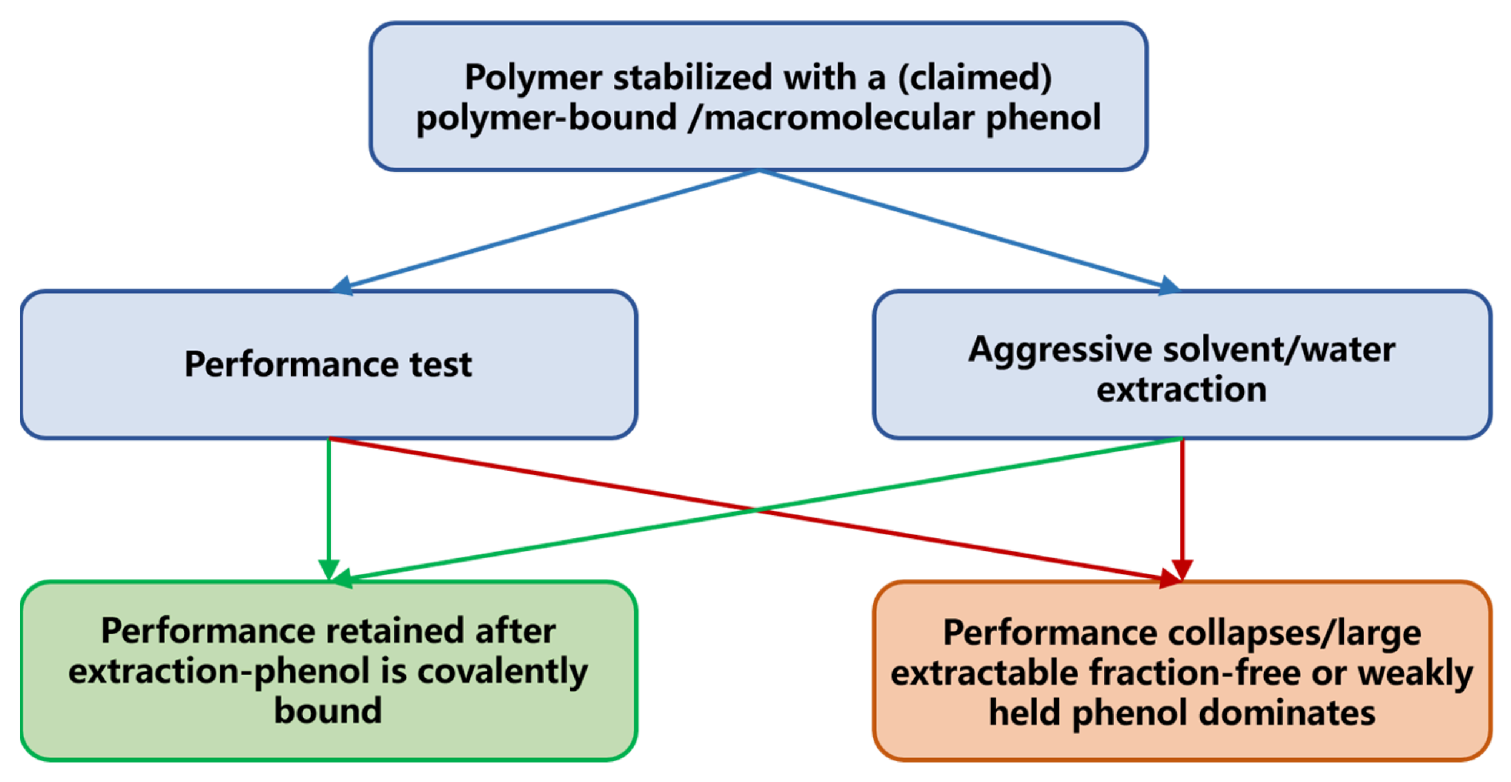
The extraction-based verification workflow for anti-migration claims: retention testing, mass-balance quantification, and structural confirmation of covalent attachment (Section 3.2).

Anti-migration claims in this field rest on a heterogeneous set of tests, which complicates cross-study comparison. Food-contact-style migration experiments expose films to simulants (ethanolic, acidic, fatty) under regulated time–temperature conditions and quantify the transferred phenol chromatographically; this is the protocol behind most of the packaging evidence discussed in Section 2.1 [10,11,13]. Solvent extraction and boiling-water tests are harsher, accelerated proxies used for pipes and geosynthetics, often coupled with mass balance and diffusion-modeling extrapolation [16,17]. Volatility is assessed by thermogravimetry, though TGA onset temperatures of the neat additive systematically overstate retention in a real matrix, where partitioning and dispersion intervene [19]. The workflow of extraction-based verification, together with its interpretive limits, is summarized schematically in Figure 4.

Two methodologically exemplary practices recur in the strongest papers reviewed in Section 4 and deserve to become field standards. The first is the before/after-extraction performance comparison. Balzadeh and Arabi showed that their ethylene–phenol copolymers retained, and in fact slightly exceeded, their oxidation induction times after solvent extraction, providing strong evidence that the phenolic functionality remains associated with the polymer [29]. The grafted-antioxidant study used the same logic in reverse, showing that the bound phenol remains functional after extraction while physical-blend controls fail instantaneously [21]. The second practice is direct quantification of the leached fraction under multiple extractants. In the hyperbranched polyglycerol (HbPG)–antioxidant work, the leached fraction was quantified directly by nuclear magnetic resonance (NMR): after 240 h, only 8% of the macromolecular phenol was extracted by hexane and 3% by water, versus more than 30% and about 9% for Irganox 1010, covering the two polarity extremes relevant to service [39]. A recurring weakness elsewhere in the literature is that retention is demonstrated only qualitatively, without extracted-fraction mass balances. Routine reporting of extractable percentage alongside the oxidation induction time (OIT) would make anti-migration claims comparable across laboratories.

A caveat on interpretation is necessary. Retention of antioxidant activity after extraction demonstrates functional retention and low extractability; it does not, by itself, prove covalent attachment. Physical entanglement of long chains, low additive solubility in the extractant, restricted solvent penetration into semicrystalline matrices, strong non-covalent host interactions, and incomplete extraction can produce the same experimental signature, and several non-covalent systems reviewed here (Section 4.3.2 and Section 5.7) do survive aggressive extraction protocols. Equally, an increase in oxidation induction time after extraction should not be read as proof that less effective species were removed unless the extracted fraction is chemically identified. Direct verification of covalent binding therefore requires structural evidence: spectroscopic confirmation on the purified polymer (NMR, Fourier-transform infrared (FTIR) spectroscopy), chromatographic fractionation, or chemical identification of the extracted and retained fractions. The studies reviewed in Section 4 are most convincing where extraction tests are paired with such structural characterization.

### 3.3. Methods for Assessing Stabilization Performance

Oxidation resistance itself is most commonly ranked by OIT or onset temperature from differential scanning calorimetry (DSC), and by oven aging with periodic mechanical or spectroscopic (carbonyl index) follow-up. These methods are sensitive and convenient but are also strongly influenced by additive volatility at test temperature, precisely the property under study, so comparisons between monomeric and macromolecular phenols can be confounded unless retention is verified independently. Chemiluminescence (CL), which detects the weak photon emission accompanying peroxy-radical termination, offers a far more sensitive probe of the earliest oxidation stages and has been developed into a versatile tool for polyolefin oxidation studies [70]. CL intensity has been correlated with oxygen uptake and peroxide concentration, though the relationship depends on polymer type, stabilizer, and sample geometry, requiring care in quantitative interpretation [71,72]. Applied to polypropylene and other polymer systems, CL further resolves photo- and thermo-oxidation under diverse conditions, including heterogeneous and filled formulations, which is relevant to how degradation propagates when stabilizers are lost from the surface layer [73,74,75]. Notably, CL has recently been applied to evaluate the recyclability of polypropylene, illustrating how sensitive oxidation monitoring supports the circular-economy applications discussed in Section 5 [57].

Across all these methods, the strongest studies in the reviewed literature are those that pair a retention measurement (extraction, migration, or spectroscopic tracking of the phenol) with a performance measurement (OIT, oven aging, or CL) on the same samples; either measurement alone leaves the anti-migration claim underdetermined. A final caveat concerns statistics: the quantitative values quoted throughout this review are almost exclusively single measurements, and the original studies rarely report replicate statistics or measurement uncertainty. Comparative phrases such as ‘threefold lower’ or ‘sevenfold difference’ should therefore be read as values reported under the stated conditions, not as statistically established effects.

### 3.4. Structure–Retention–Performance Relationships: What Is Missing

Despite two decades of activity, no study has yet mapped phenol retention and stabilization efficiency across architectures under a single standardized protocol. Quantities that would enable rational design (extracted fraction versus molar mass of the carrier, phenol utilization efficiency in the solid state, retention under combined hydrolytic and thermal stress) are reported sporadically and under incompatible conditions. This methodological fragmentation, more than any shortage of new chemistries, is what currently prevents the field from converging on design rules.

## 4. Molecular Architectures of Polymer-Bound and Macromolecular Hindered Phenolic Antioxidants

**Figure 5:**
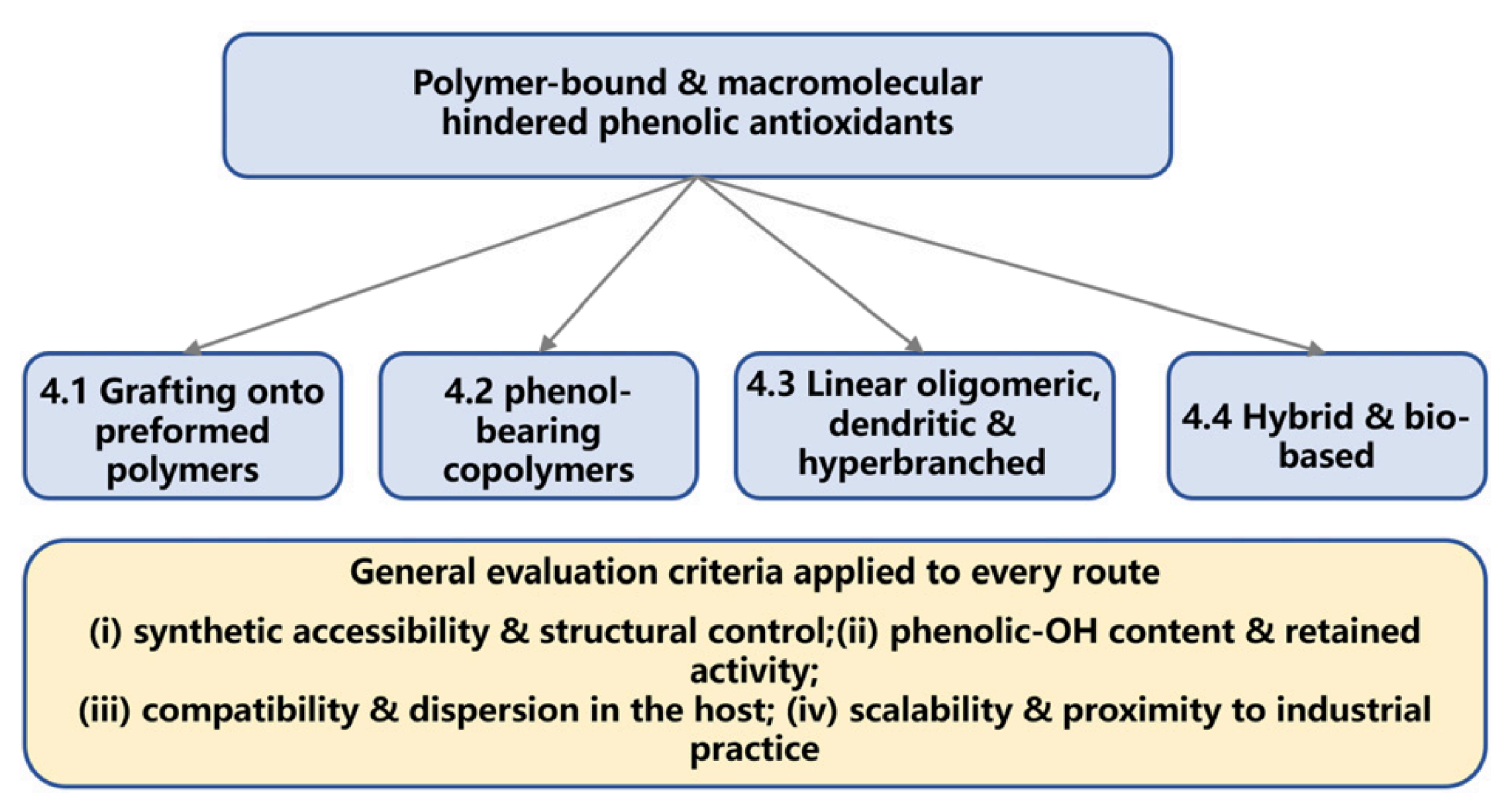
Classification of polymer-bound and macromolecular hindered phenolic antioxidants into the four architecture families reviewed in Section 4.1, Section 4.2, Section 4.3 and Section 4.4, organized by the single primary axis of molecular architecture. Application sectors are treated separately in Section 5; the lower panel lists the general evaluation criteria applied to every family.

Macromolecular hindered phenols reported to date span a wide range of attachment chemistries (post-polymerization grafting, direct copolymerization, stepwise or one-pot dendritic/hyperbranched synthesis, supramolecular assembly, and hybridization onto inorganic or biological scaffolds) and a corresponding range of molecular architectures (linear grafts, side-chain copolymers, dendritic or hyperbranched structures, nanoparticulates, and bio-based macromolecules). In this review, the classification is based on a single primary axis—the molecular architecture (Figure 5)—because topology is an intrinsic material property that does not depend on the intended application; the attachment chemistry is discussed within each family as the synthetic route that generates the architecture, not as a second classification axis. This yields four families (Figure 5, upper panel): grafting onto preformed polymers (Section 4.1), phenol-bearing copolymers formed by direct copolymerization (Section 4.2), linear oligomeric, dendritic, and hyperbranched architectures (Section 4.3, in which the linear oligomeric phenols serve as the industrial baseline within this family), and hybrid and bio-based scaffolds (Section 4.4). Systems developed for elastomer hosts are discussed within their topological families—a grafted polybutadiene-bound phenol in Section 4.1, a star-shaped thiol–ene oligomer in Section 4.3.2, and phenol-functionalized graphene in Section 4.4—while the sector-specific requirements of elastomers are addressed in Section 5.4. To make the comparison across these families useful rather than merely descriptive, each family is assessed against the same four general criteria (Figure 5, lower panel): (i) structural control, including synthetic accessibility; (ii) phenol content and retained activity, i.e., the phenolic hydroxyl content and the retention of intrinsic activity after immobilization; (iii) compatibility and dispersion in the intended host polymer; and (iv) scalability, i.e., proximity to industrial practice. Within each subsection, at least one representative study is examined in quantitative depth, because the published performance claims differ so widely in test protocol that only study-level analysis reveals what the numbers actually support; the test methods and their caveats established in Section 3 serve as the interpretive lens throughout. A cross-architecture assessment is given in Section 4.5.

### 4.1. Grafting onto Preformed Polymers via Reactive Processing

**Figure 6:**
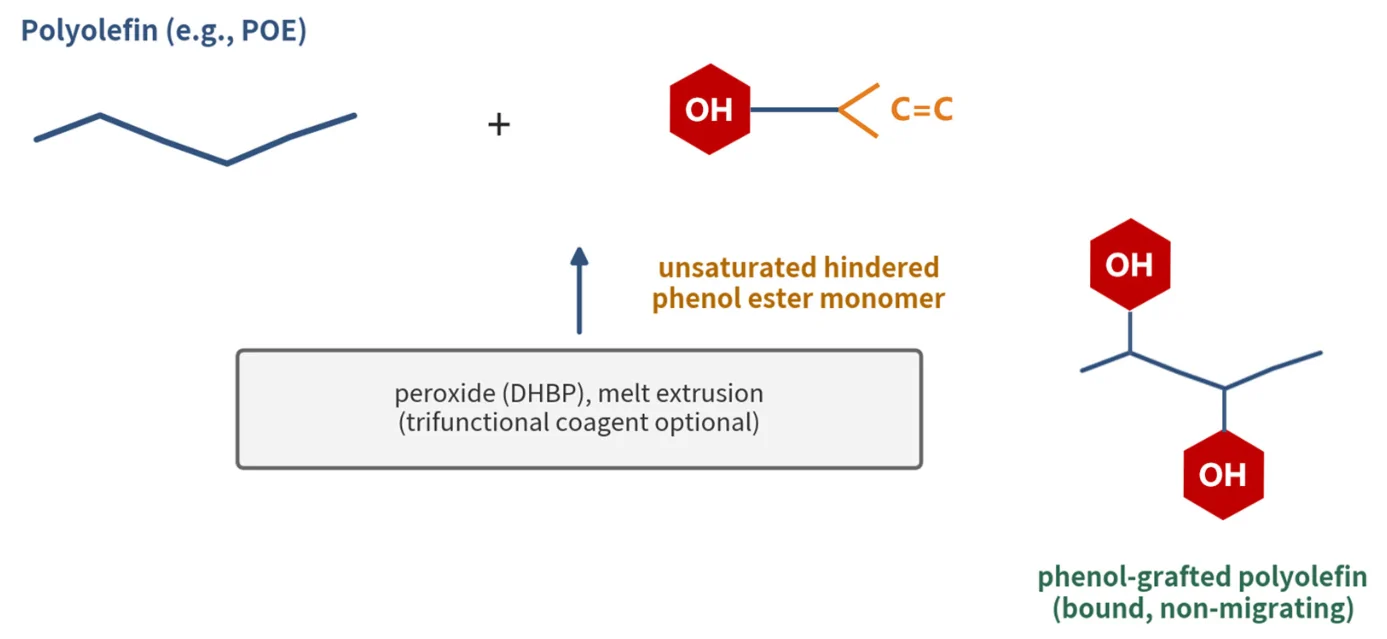
Reactive grafting of an unsaturated hindered phenol ester onto a preformed polyolefin during peroxide-initiated melt processing (Section 4.1).

The most industrially direct route is the covalent attachment of a polymerizable or otherwise reactive hindered phenol onto a preformed polyolefin during melt processing (Figure 6). The chemistry is older than the recent literature suggests. A persistent limitation of this route is the competition between grafting and homopolymerization of the functional monomer, which keeps grafting efficiency low. Zheng and co-workers showed that tea polyphenols, natural molecules carrying multiple phenolic units, act as an efficient grafting coagent in the dicumyl-peroxide-initiated melt grafting of glycidyl methacrylate onto polypropylene: added at only 0.5–3 wt%, the polyphenol provides multiple reactive sites that capture polypropylene backbone radicals and markedly raise the grafting degree [27]. A solvent-free alternative to melt grafting has recently been demonstrated by mechanochemistry. Di Maro and co-workers covalently linked properly designed butylated-hydroxytoluene antioxidants (and hindered-amine light stabilizers) to maleic-anhydride-grafted polypropylene in a ball mill under mild, solvent-free conditions, quantitatively evaluating the grafting outcome and showing that the resulting polypropylene-based macromolecular stabilizers combine outstanding thermal behavior with permanent anchoring of the stabilizer [26]. Related maleimide-functionalized antioxidants have also been formulated into polymeric antioxidant systems [76].

Representative recent studies in this family, discussed here as illustrative rather than exhaustive, come largely from Arabi and co-workers, who have systematically developed the ester-monomer variant. Unsaturated hindered phenol esters were grafted onto polyolefin elastomers by peroxide-initiated reactive processing, and the grafted products showed antioxidant behavior clearly superior to their physical-blend counterparts [21]. Covalent immobilization of phenolic antioxidants on ethylene copolymers was shown to provide efficient long-term stabilization of polypropylene, with the graft used as a masterbatch-type additive [22]: at 3 wt% in unstabilized PP powder, the bound-antioxidant blends reached oxidation induction times of 11.5 min (polyolefin elastomer (POE) carrier) and 9.5 min (ethylene–vinyl acetate (EVA) carrier, despite bearing twice the phenol content), versus only 1.3 min for an equivalent free phenolic acid, and the bound systems retained their OIT through ethanol extraction while PP stabilized with Irganox 1010 fell to 4.2 min after 3 days. In a further refinement, the hindered phenol was covalently attached to surface-functionalized polypropylene, enhancing thermo-oxidative stability while localizing the antioxidant where oxidation initiates [23]. In melt rheology, a sensitive probe of chain scission, a PP/PP-bound-phenol blend at 3:1 showed no measurable viscosity decay over a 100 min test in the molten state, and retained this stability after successive extraction in acetone at 50 °C and hexane at 70 °C for 3 days each, conditions under which the conventionally stabilized control degraded rapidly.

The first of these studies [21] rewards closer examination because it quantifies three structure–processing variables that every practitioner of this route must choose. The first variable is the phenolic acid precursor. Monomeric antioxidants were prepared by esterifying either 3,5-di-tert-butyl-4-hydroxybenzoic acid or 3-(3,5-di-tert-butyl-4-hydroxyphenyl)propionic acid with unsaturated fatty alcohols, and the propionic-acid esters exhibited significantly longer oxidation induction times, with the oleyl ester of the propionic-acid series reaching an OIT of almost 43 min in blend tests. This is consistent with the well-known deactivating conjugation of the carboxyl group directly attached to the aromatic ring in the benzoic series. The second variable is the initiator system. Among methyl ethyl ketone (MEK) peroxide, dicumyl peroxide (DCP), and 2,5-dimethyl-2,5-di(tert-butylperoxy)hexane (DHBP), DHBP was the most effective at increasing the amount of antioxidant grafted: grafted POE prepared with DHBP showed oxidation induction times two to three times longer than those made with DCP or MEK peroxide, and a DHBP/Sn(Oct)2 redox system raised the graft content further, delaying the induction time of the grafted elastomer to almost 10 min. The third observation is the most diagnostic. The phenolic moiety remained functional after solvent extraction of the grafted polymer, whereas both the unstabilized polymer and a commercially stabilized physical blend were degraded essentially instantaneously under the same oxidative test. This before/after-extraction comparison is the most direct demonstration that the activity resides in bound, not residual free, phenol, and it is a test protocol that surprisingly few subsequent papers in this field have adopted as a standard.

Two variations illustrate the versatility of the reactive route. Peroxide-initiated chemical modification of polyolefins has been explored with the aim of creating latent antioxidants, that is, grafted precursor structures that survive processing and liberate the active phenol subsequently, decoupling stabilizer attachment from stabilizer action [24]. A technically important special case is peroxide-crosslinked polyethylene for cable insulation, where the antioxidant must survive the crosslinking step or participate in it benignly. Reactive antioxidants designed to be grafted during peroxide crosslinking have been shown to meet this requirement [25]: retention of the graftable phenol/hindered-amine stabilizer (HAS) system in PEXa pipes exceeded 85% after stringent dichloromethane extraction, far above the conventional Irganox 1076/HAS formulation, and in hydrostatic pressure testing at 115 °C and 2.5 MPa the pipes stabilized with the graftable system outlasted the conventional formulation, which failed in just over one year (as reported; replicate statistics were not provided).

Critically assessed, grafting onto preformed polymers is the closest of all reviewed routes to existing industrial practice: it is a one-step, solvent-free, continuous process using standard extrusion equipment. Its weaknesses are equally characteristic, and the deep-dive study above makes them concrete: because grafting competes with homopolymerization of the monomeric phenol and with peroxide-induced chain scission or crosslinking of the matrix, the product is typically a mixture of grafted polymer, unreacted migratable monomer, and phenol homopolymer; graft content must be pushed by initiator optimization rather than being set by stoichiometry; and the attainable phenolic hydroxyl density is bounded by the graft level, well below what purpose-built architectures can deliver. Structural control is inherently poor: the product is a distribution of graft lengths and sites rather than a defined molecule.

Grafting is equally applicable to unsaturated elastomer backbones, which represent among the most demanding retention environments: the mechanistic study of a polyhydroxylated-polybutadiene-bound thiobisphenol (PHPBT-b-TPH) in natural rubber vulcanizates [45] provides the quantitative depth for this route. At a loading of only 1 phr, the macromolecular antioxidant delivered thermo-oxidative aging resistance clearly superior to the same loading of the corresponding low-molar-mass thiobisphenol TPH: after aging at 100 °C for 168 h, the vulcanizate containing PHPBT-b-TPH retained 43.6% of its tensile strength and 58.6% of its elongation at break, against 35.6% and 54.5%, respectively, for the TPH-containing control. The absolute retentions also deserve comment: even the best sample lost more than half of its initial tensile strength under this accelerated protocol, underlining how severe thermo-oxidative aging of NR is and how much headroom remains for improved stabilizers. The mechanistic finding of the same study, that both the thioether and the urethane groups possess intrinsic antioxidative ability and act synergistically with the hindered phenol, rationalizes why the macromolecular design outperforms an equimolar phenol comparison: the backbone is not an inert carrier but a co-stabilizer. This multi-functionality principle is one of the clearest design lessons the elastomer literature offers to the wider field.

These studies are significant beyond their specific chemistry: they demonstrate that matching the macromolecular antioxidant to the host—a flexible, polar-modified backbone for a flexible, lightly polar rubber—is as important as maximizing phenol content. At the same time, the elastomer literature exposes a general weakness of the field: phenolic hydroxyl content per unit mass of these macromolecules is necessarily lower than that of monomeric phenols, so equal radical-scavenging capacity requires higher additive loadings, with potential effects on processing, plasticization, and crosslink chemistry that are rarely quantified systematically.

### 4.2. Direct Copolymerization of Phenol-Bearing Monomers

**Figure 7:**
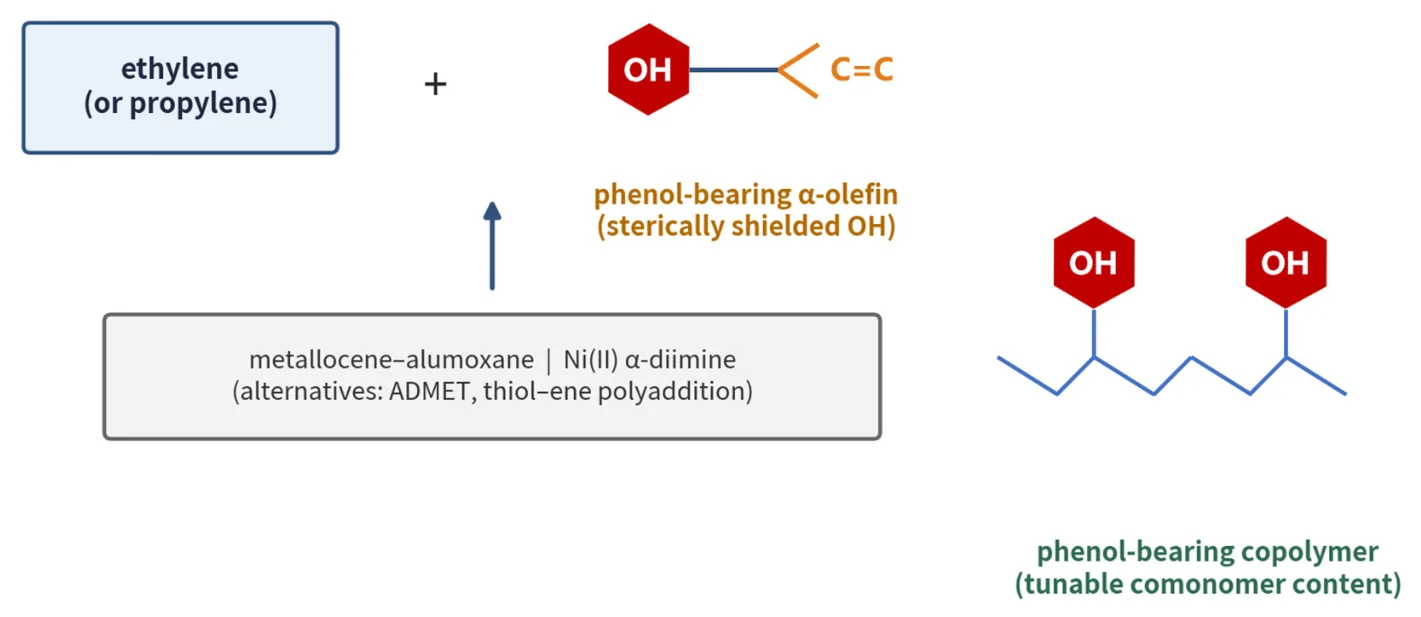
Direct copolymerization of ethylene with a sterically shielded, phenol-bearing α-olefin (Section 4.2).

Building the hindered phenol into the chain during polymerization offers the highest degree of structural control of any route in this section, and it is also the most counterintuitive. Hindered phenols are chain-breaking radical scavengers by design. They quench the very carbon- and oxygen-centered radicals on which chain-growth polymerization depends, and their hydroxyl groups coordinate to the electrophilic metal centers of olefin-polymerization catalysts. A phenol-bearing monomer is therefore, in the general case, a retarder, an inhibitor, or a catalyst poison rather than a comonomer. Direct copolymerization becomes feasible only under narrowly defined conditions: the phenolic hydroxyl must be sterically shielded, and the polymerizable double bond must be kept at a distance from the aromatic ring. Much of the literature of this family can be read as different solutions to these two constraints. That they can indeed be solved was demonstrated across a surprisingly broad catalytic toolbox. Chung and co-workers, for example, used direct copolymerization to build cross-linkable hindered-phenol moieties into polypropylene, and the covalently anchored phenols gave the copolymer long-term stability under elevated-temperature aging [32]. Remarkably, the phenolic comonomer raised the initial polymerization rate by up to almost threefold over the two chiral bridged zirconocene catalysts while leaving Cp2ZrCl2 activity essentially unchanged, an early quantitative hint that a properly shielded phenol need not poison metallocene centers. Yang and co-workers recently achieved the direct copolymerization of ethylene with antioxidant polar comonomers using a phosphophenol nickel catalyst, yielding intrinsically antioxidant, non-migratory UHMWPE of 1–5 million molar mass [33]. The copolymers showed no detectable oxidation exotherm within 60 min at 200 °C, conditions under which commercial UHMWPE oxidized rapidly, and after 24 h of reflux extraction in acetone at 80 °C the covalently incorporated phenol was reported to be retained essentially completely, whereas a physically blended vitamin E control was almost quantitatively leached out. The same group later showed that such polypropylene-bonded hindered phenol copolymers can simply be blended into commercial polypropylene: cocrystallization between the PP matrix and the PP-HP copolymer distributes the bound phenol homogeneously in the amorphous domains, and the resulting blends show a thermal–oxidative stability nearly proportional to the bound-phenol content, remaining stable far beyond the few minutes that commercial PP products survive at 210 °C in air [77]. The route is illustrated schematically in Figure 7.

Coordination catalysis is not the only way to place a phenol on a growing chain: step-growth polymerizations avoid metal centers altogether and are therefore intrinsically tolerant of phenolic functionality. Acyclic diene metathesis (ADMET) polymerization [34] is an instructive case in which a first-generation cinnamate-type macromonomer gave only an 18 min OIT in PP at 185 °C, while redesigning the pendant to the same propionate phenol used in Irganox 1010 raised stability so sharply that measurement had to move to 200 °C, where the macromolecular system oxidized after 34 min against 41 min for Irganox 1010 itself. Thiol–ene polyaddition offers a second step-growth route [78], which supplied a macromolecular thioether synergist: PP containing 0.55 wt% of the ADMET phenol plus 0.27 wt% of the polythioether began to oxidize only after 339 min at 185 °C, versus 115 min for nearly double the phenol alone, 9 min for the synergist alone, and 57 min for an Irgafos 168 reference, a rare quantitative dissection of primary/secondary antioxidant synergism in a fully macromolecular formulation.

Representative studies discussed in depth below illustrate the range of this family rather than exhaust it. Chung and co-workers built a family of polypropylene-backbone stabilizers (PP-HP, with random and tapered microstructures) by Steglich esterification of hydroxylated polypropylene with a carboxylic-acid-functionalized hindered phenol. The PP chain degradation temperature increased monotonically with phenol content, several PP-HP copolymers were significantly more thermo-oxidatively stable than commercial PP containing a monomeric phenol stabilizer, and the effect persisted in PP/PP-HP blends. The copolymer thus works as a masterbatch, an effect attributed to its compatibility and cocrystallization with the PP host giving a uniform phenol distribution. The material was then demonstrated in metallized polymer film capacitors, a demanding dielectric application in which additive migration is especially damaging [28]. Balzadeh and Arabi prepared polyethylene copolymers carrying ancillary phenolic antioxidant groups and analyzed their chemical composition and thermo-oxidative stability in detail, establishing the copolymer approach as a route to polyolefin stabilizers with no detectable migration under the reported test conditions [29]. The same group subsequently used a tailor-made nickel(II) α-diimine catalyst to produce dendritic, phenolic-antioxidant-functionalized polyethylene directly by copolymerization, obtaining an additive with no detectable release under the reported extraction protocols, whose topology is set catalytically rather than by post-modification [30]. The reported metrics are impressive: about 0.6 wt% comonomer incorporation at roughly 80% conversion, versus at most about 30% for Ziegler–Natta and group-IV metallocene catalysts; oxidation induction times tunable over 2–36 min by the phenolic comonomer content, against at most 18 min for the other catalyst families at comparable incorporation; and, decisive for the non-releasing claim, an essentially unchanged OIT after 15 days of extraction in boiling chloroform, under conditions reported to wash out BHA, Irganox 1076, and Irganox 1010 from polyethylene almost completely within 5 days.

**Figure 8:**
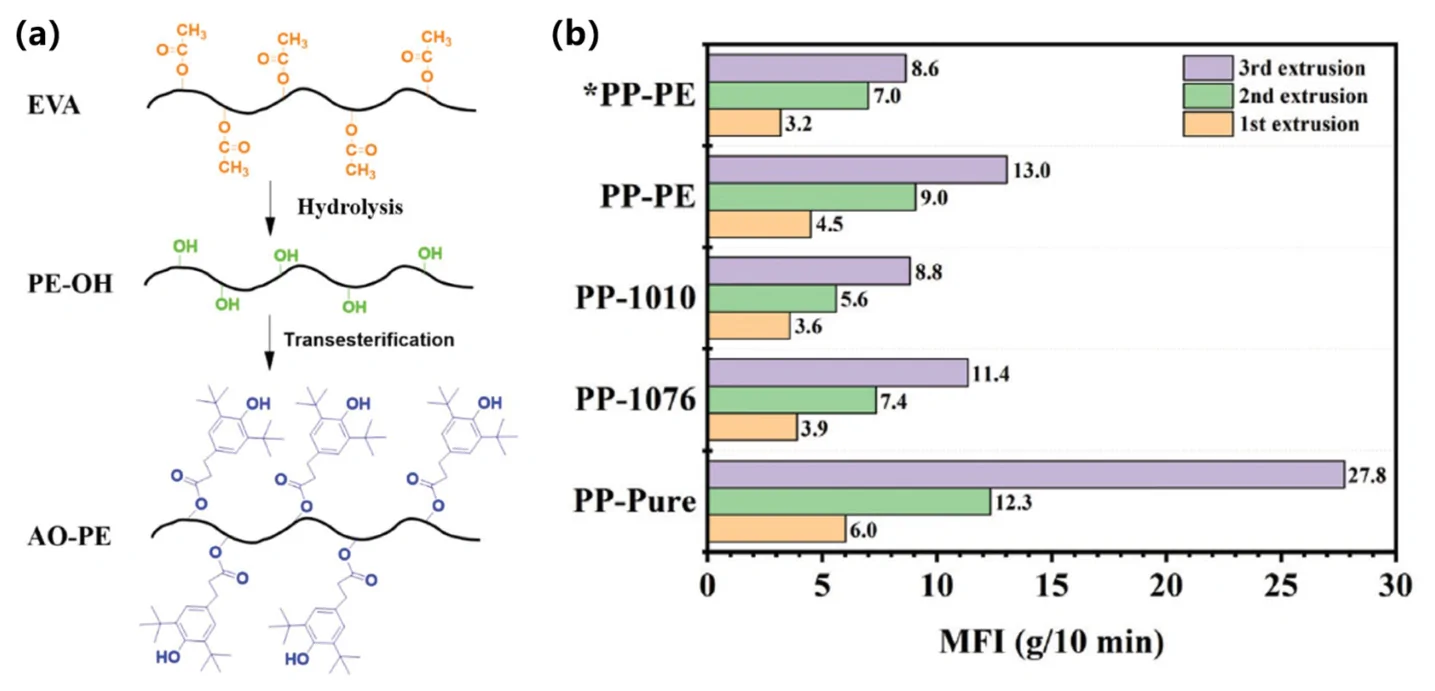
(**a**) Scheme of synthesis of PE-OH and AO-PE. (**b**) Melt flow index of polypropylene stabilized with a polyethylene-based macromolecular hindered-phenol antioxidant (PP-PE) versus Irganox 1010, Irganox 1076, and an unstabilized control, over three consecutive extrusion passes. * PP-PE: dosage of the macromolecular antioxidant raised to about three times the standard 1000 ppm level. Reproduced with permission from ref. [31]. Copyright 2020, Wiley-VCH.

A complementary post-modification of a commercial polymer is also worth noting. Hydrolysis of ethylene–vinyl acetate copolymer followed by transesterification with methyl 3-(3,5-di-tert-butyl-4-hydroxyphenyl)propionate converts an off-the-shelf EVA into a polyethylene-backbone antioxidant bearing hindered phenol side groups. The products are more thermally stable in air than the monomeric standards Irganox 1076 and 1010, and their effectiveness in multiply extruded polypropylene, tracked by melt-flow index, increases with the molar mass of the antioxidant backbone [31]. In that melt-flow-index comparison (Figure 8), unstabilized polypropylene climbs from 6.0 to 27.8 g/10 min over three extrusion passes, whereas the polyethylene-bound phenol limits the third-pass value to 8.6 g/10 min, below both monomeric benchmarks (11.4 for Irganox 1076 and 8.8 for Irganox 1010), because the phenol-bearing chain can neither volatilize nor exude during repeated melt processing.

The thermal-analysis study of the ethylene–phenol copolymers [29] is the most instructive single paper in this family, for two reasons. Methodologically, it establishes a practical assay for bound-phenol content. TGA and derivative thermogravimetry (DTG) curves of the copolymers show two distinct degradation events under oxygen: one near 250 °C, assigned to dissociation of the ether linkage tethering the phenol, and a second between 450 and 500 °C for the polyethylene backbone. The weight loss of the first event scales with the phenol incorporation and can therefore serve as an antioxidant-content index, and the oxidation induction time increases steeply with this index, giving a direct composition–performance relationship across copolymers made under different polymerization conditions. Mechanistically, the key experiment is again an extraction test. After solvent extraction, the copolymers showed higher OIT values than before, indicating that the antioxidant functionality remains associated with the polyethylene carrier and that no extractable low-molar-mass antioxidant accounts for the measured stability; taken together with the TGA evidence for a tethered phenol, covalent bonding is the most plausible interpretation. The slight increase in OIT after extraction is consistent with, but does not by itself prove, the removal of less effective oligomeric species. The companion dendritic-PE study [30] adds the monomer-design rules that make such copolymerization feasible at all. The phenolic monomer (2,6-di-tert-butyl-4-(dec-9-en-1-yloxy)phenol) was engineered so that the hydroxyl group is sterically shielded to minimize poisoning of the Ni(II) α-diimine active centers, while a long alkenyl spacer separates the polymerizable double bond from the phenolic ring. These two constraints, protecting the catalyst from the phenol and protecting the phenol from the polymerization, define the synthetic design space of the whole copolymerization family.

The decisive advantage of copolymerization is that the antioxidant can be the same polymer species as the host: a phenol-bearing polyethylene blended into polyethylene presents no chemical interface, so compatibility is intrinsic and extraction requires removal of a covalently bound macromolecule. Molar mass, branch topology, and comonomer content are controlled at the synthesis stage, giving reproducible phenol densities; the data above show this reproducibility can be monitored by a simple TGA assay. The barriers are equally clear: the phenolic functionality must survive or be masked during catalytic polymerization, the route requires organometallic polymerization capability that lies far outside additive compounding, and the approach is essentially restricted to polyolefin hosts. Cost and scale-up of specialty copolymers remain the main obstacles to commercialization.

### 4.3. Linear Oligomeric, Dendritic, and Hyperbranched Architectures

#### 4.3.1. Linear Oligomeric Phenols: The Industrial Baseline

Between monomeric phenols and true macromolecular designs sits a commercially important class that is often overlooked in the academic literature: linear oligomeric hindered phenols with molar masses roughly in the 500–1500 g mol^−1^ range. The longest-established example is the butylated reaction product of p-cresol with dicyclopentadiene (CAS 68610-51-5), a hydrogenated oligomeric polyphenol resin marketed for decades under trade names such as Wingstay L and Lowinox CPL. According to the manufacturers’ technical data sheets (Synthomer, formerly Eliokem, for Wingstay L [79]; SI Group, formerly Addivant, for Lowinox CPL [80]), its number-averaged molar mass is only about 600–700 g mol^−1^, yet this is sufficient to suppress volatility and extraction relative to monomeric phenols—the data sheets emphasize resistance to extraction by washing and boiling—which is why it remains a workhorse in the stabilization of natural rubber, styrene–butadiene elastomers, latex, and polyolefins, with food-contact clearances including FDA 21 CFR 178.2010 [81]. Related industrial families include alkylidene-bridged polyphenols and higher-functionality ester phenols whose size, rather than any covalent attachment, provides migration resistance. They are the purest industrial embodiment of the transport-retardation-by-size mechanism discussed in Section 3.1. A historical assessment of the additives industry adds a sobering note [82]: according to that industry account, oligomeric and polymeric phenolic structures have repeatedly been developed, including siloxane-backbone phenols (Silanox) and polyphenols derived from enzymatic or oxidative polymerization (Polnox), but most were withdrawn, and their commercial success has remained far more limited than that of oligomeric hindered-amine light stabilizers; we are not aware of primary industrial documentation for the current status of these withdrawn products. This track record sets the industrial baseline against which the more elaborate architectures of Section 4.3.2 and Section 4.4 must be measured. Any new macromolecular phenol competes not only with monomeric Irganox-type esters on activity, but also with cheap, proven oligomeric resins on cost per unit of retained stabilization.

#### 4.3.2. Dendritic and Hyperbranched Syntheses

**Figure 9:**
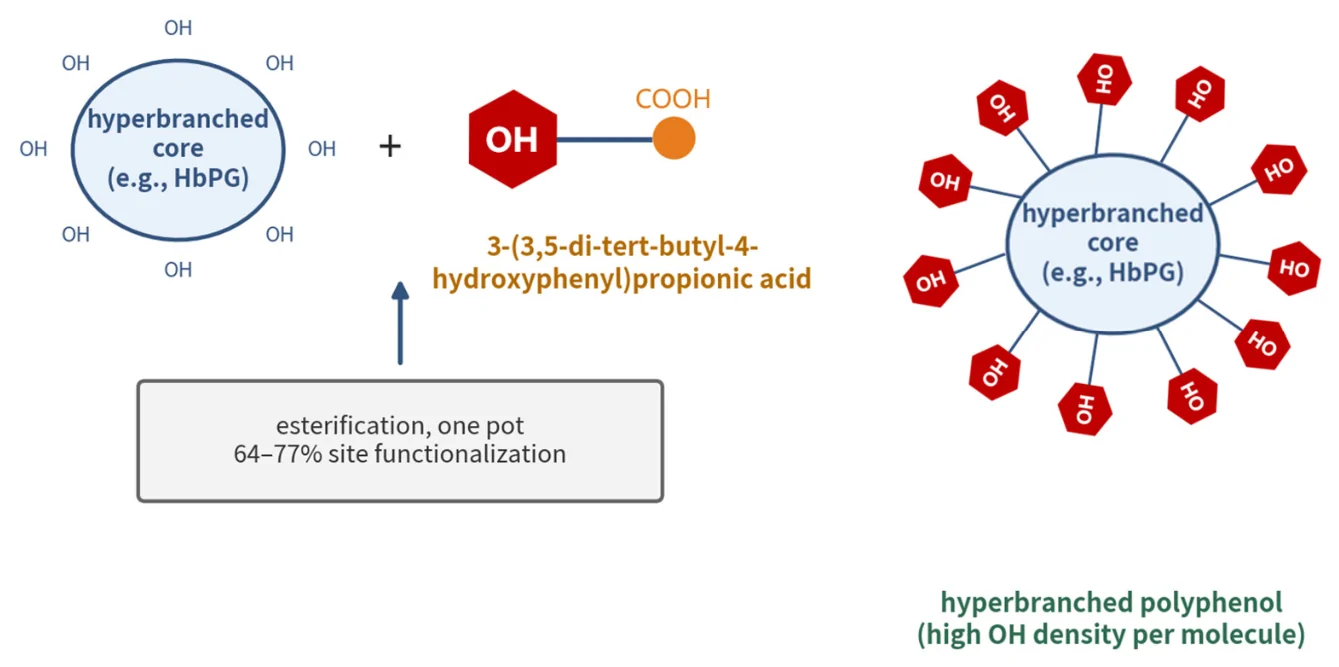
One-pot synthesis of hyperbranched polyphenol antioxidants by esterification of a hyperbranched polyglycerol core with a hindered phenolic acid (Section 4.3.2).

Dendritic and hyperbranched phenols aim to maximize the number of phenolic groups carried per molecule, and hence per unit of diffusing mass. Dendritic phenolic antioxidants of three generations, built from 2,2-bis(hydroxymethyl)propionic acid branching units with 3-(3,5-di-tert-butyl-4-hydroxyphenyl)propionic ester chain ends, established the generational scaling of phenol content with stabilization performance [35]. Thermogravimetry showed all of the functionalized dendrons to be more thermally stable than the commercial antioxidants BHT, Irganox L135, and Irganox L57, and pressurized-DSC screening in a lubricant base oil at only 0.5 wt% ranked the dendritic additives ahead of these commercial references. Hindered phenols bridged by aliphatic diamines of different chain length carry two phenolic units per molecule; in polypropylene and linear low-density polyethylene (LLDPE) they improved melt-flow retention and oxidation induction time, and at equal phenolic-hydroxyl concentration the antioxidant activity and mechanical-property retention increased with bridge length [36]. Dendrimer-bridged hindered phenols of the 1.0 generation, in which several phenolic units are attached to a dendritic core through bridges of different length, connect solution activity to in-polymer performance [37]. Their 2,2-diphenyl-1-picrylhydrazyl (DPPH) radical-scavenging ability decreased as the bridge lengthened, a slow-kinetics scavenging behavior, yet in LLDPE and polypropylene the oxidation induction times and aging coefficients increased with bridge length, and the dendritic phenols showed excellent processing stability and thermo-oxidative aging resistance, performing better in LLDPE than in polypropylene. Hyperbranched macromolecule-bridged hindered phenols, in which phenolic units are linked by a hyperbranched core, similarly showed high oxidative stability [38]. Hyperbranched poly(ethyleneimine)-based macromolecular antioxidants proved effective in both polyethylene and polypropylene, with measurably lower leaching than the tetrafunctional Irganox 1010 [83]: after 70 h in n-hexane at room temperature, Irganox 1010 was reported to be almost completely washed out of the PP films whereas the hyperbranched PEI conjugates showed little detectable loss. No extraction at all was detected in water at 65 °C over 27 h, but this result should not be read as evidence of binding, because even the free Irganox 1010 control showed no detectable extraction under these conditions: the hot-water test does not discriminate between bound and free phenols, and the discriminating retention evidence comes from the hexane test. The one-pot esterification route used to build such hyperbranched polyphenol antioxidants is illustrated in Figure 9.

Among the dendritic and hyperbranched systems surveyed above, the hyperbranched polyglycerol (HbPG) nanoparticle antioxidants of Kasza, Iván and co-workers [39] provide the most complete quantitative dataset available for this family and repay detailed reading. Esterification of HbPG cores of different molar masses with 3-(3,5-di-tert-butyl-4-hydroxyphenyl)propionic acid (Figure 9) achieved functionalization of 64–77% of the available hydroxyl groups. The synthetic chemistry thus converts a majority, but not all, of the scaffold sites, and the residual hydroxyls remain as polar groups that must be considered in compatibility. The conjugates had polydispersities of 1.33–1.66, typical of one-pot ring-opening multibranching polymerization and a reminder of the polydispersity price paid for synthetic simplicity relative to stepwise dendrimers. This one-pot route is synthetically simple, but it has so far been demonstrated only at laboratory scale, and its industrial scalability remains to be established. Their glass transition near 40 °C and thermal stability up to about 300 °C mean they are processable liquids that survive standard polyolefin and poly(vinyl chloride) (PVC) processing temperatures. In poly(vinyl chloride), the HbPG-based macromolecular antioxidants raised the oxidation induction time of unstabilized PVC (about 400 s) by a factor of roughly 2.5–3, a stabilizing efficiency close to the tetrafunctional industrial standard Irganox 1010, and markedly suppressed HCl elimination and degradative chain scission. The critical discriminator, however, was retention, and in extraction tests the quantitative gap was large. After 240 h, only 8% of the HbPG-bound phenol was extracted by hexane and 3% by water, versus more than 30% and about 9%, respectively, for Irganox 1010. This is roughly threefold lower leaching in both media as reported, supporting the anti-migration design hypothesis in a polar host. Equally instructive is the contrast embedded in this literature: for dendrimer-bridged hindered phenols, the DPPH-scavenging activity in solution decreases as the bridge between core and phenol lengthens, whereas the oxidation induction time and aging coefficient in polyolefins increase [37], a reminder that intrinsic activity in solution assays does not set the performance ranking in a solid, nonpolar host, where dispersion and segmental mobility become limiting.

Although polyolefins are the natural target of this family, dendritic phenols have also been carried into more polar hosts: a non-aromatic dendritic antioxidant bearing amine groups was synthesized and showed anti-aging performance in acrylonitrile–butadiene rubber [40]. Because this additive also bears secondary amine functions, its stabilizing effect cannot be assigned exclusively to hindered-phenol chemistry. A conceptually distinct variant avoids covalent attachment altogether: in layered double hydroxide nanofillers, an interlayer “fence” structure built by partial anion exchange confines an intercalated hindered-phenol antioxidant, and in polypropylene composites this confinement cut the antioxidant migration ratio to 8.35%, against 39.33% for free Irganox 1010, while raising the 50%–decomposition temperature of the additive by 58 °C, from 346 to 404 °C [41]. This confinement system is non-covalent and therefore lies outside the covalent scope of this review; it is included because it defines what physical confinement alone can achieve, and thereby clarifies what covalent binding adds beyond it. The broader potential of dendrimers and hyperbranched polymers as additives in polyolefins has been reviewed recently [42].

Critically, this family offers the highest phenolic hydroxyl density per molecule of any reviewed architecture, and dendritic structures additionally allow precise placement of synergistic functions (amine, thioether) within one molecule. However, perfectly branched dendrimers require stepwise, protection-intensive synthesis that is impractical at scale; hyperbranched analogs made in one pot are far more realistic but are polydisperse (the PDI values above quantify this), and the literature rarely quantifies how polydispersity affects performance. High polarity of dendritic cores can impair dispersion in nonpolar polyolefins, melt-viscosity effects at useful loadings are seldom reported, and supramolecular versions, while synthetically attractive, have yet to prove retention under hot, wet, or extractive service.

**Figure 10:**
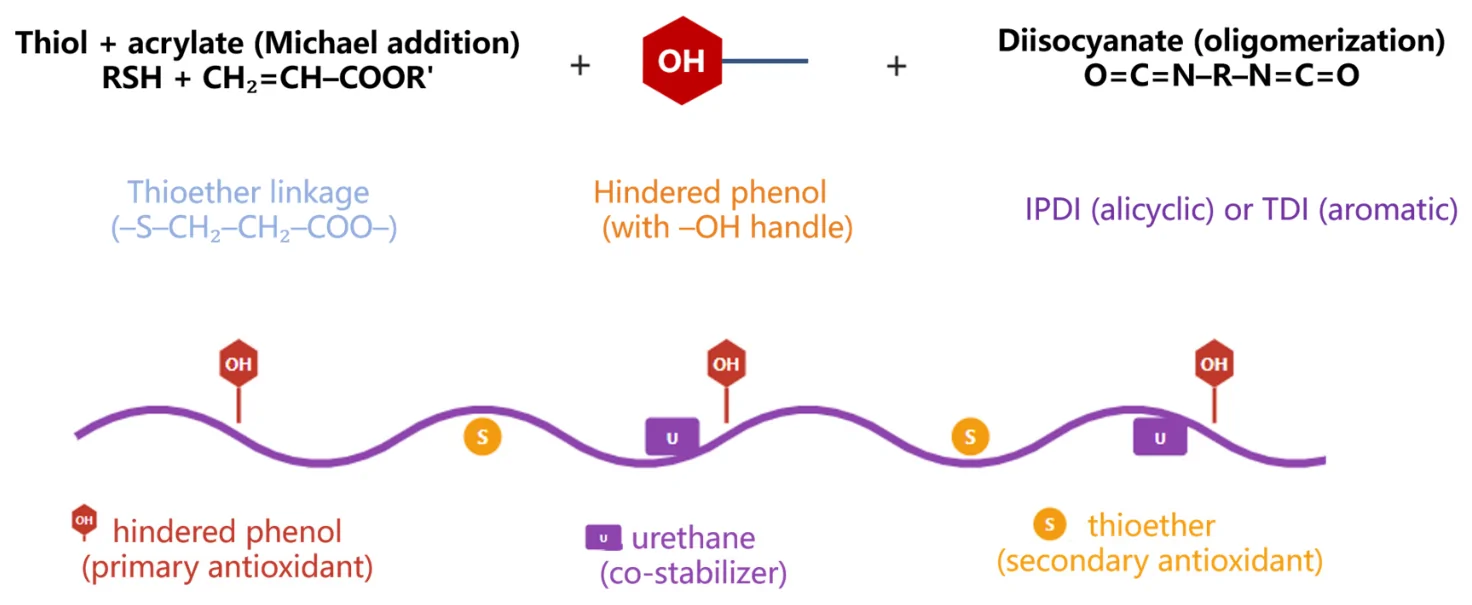
Hindered-phenol-functionalized graphene (FGE, Section 4.4) and the star-shaped thiol–ene-click macromolecular antioxidant PM (Section 4.3.2), both developed for elastomer hosts.

Star-shaped oligomers assembled by click chemistry extend this family toward elastomer hosts. A thiol–ene click route offers a modular way to oligomerize phenolic units: Liu and co-workers reacted the maleimide antioxidant N-(4-aminophenyl)maleimide (MC, 264 g mol^−1^) with pentaerythritol tetrakis(3-mercaptopropionate) to give a macromolecular antioxidant (PM, 1544 g mol^−1^, Figure 10) carrying thioether and aromatic secondary amine groups, and compared the two directly in ethylene–propylene–diene rubber (EPDM) [44]. PM reduced the interference of the additive with rubber curing and raised the crosslink density; after aging at 120 °C for 15 days, X-ray photoelectron spectroscopy (XPS) showed the surface nitrogen content of PM/EPDM rising only from 0.55% to 2.08%, against 0.69% to 4.25% for MC/EPDM, direct spectroscopic evidence that the sixfold larger molecule migrates to the surface far more slowly than its monomeric analog. Because PM carries thioether and aromatic secondary amine groups in addition to phenolic units, its performance illustrates deliberate multifunctional design rather than pure hindered-phenol chemistry.

### 4.4. Hybrid, Bio-Based, and Nanostructured Macromolecular Phenols

**Figure 11:**
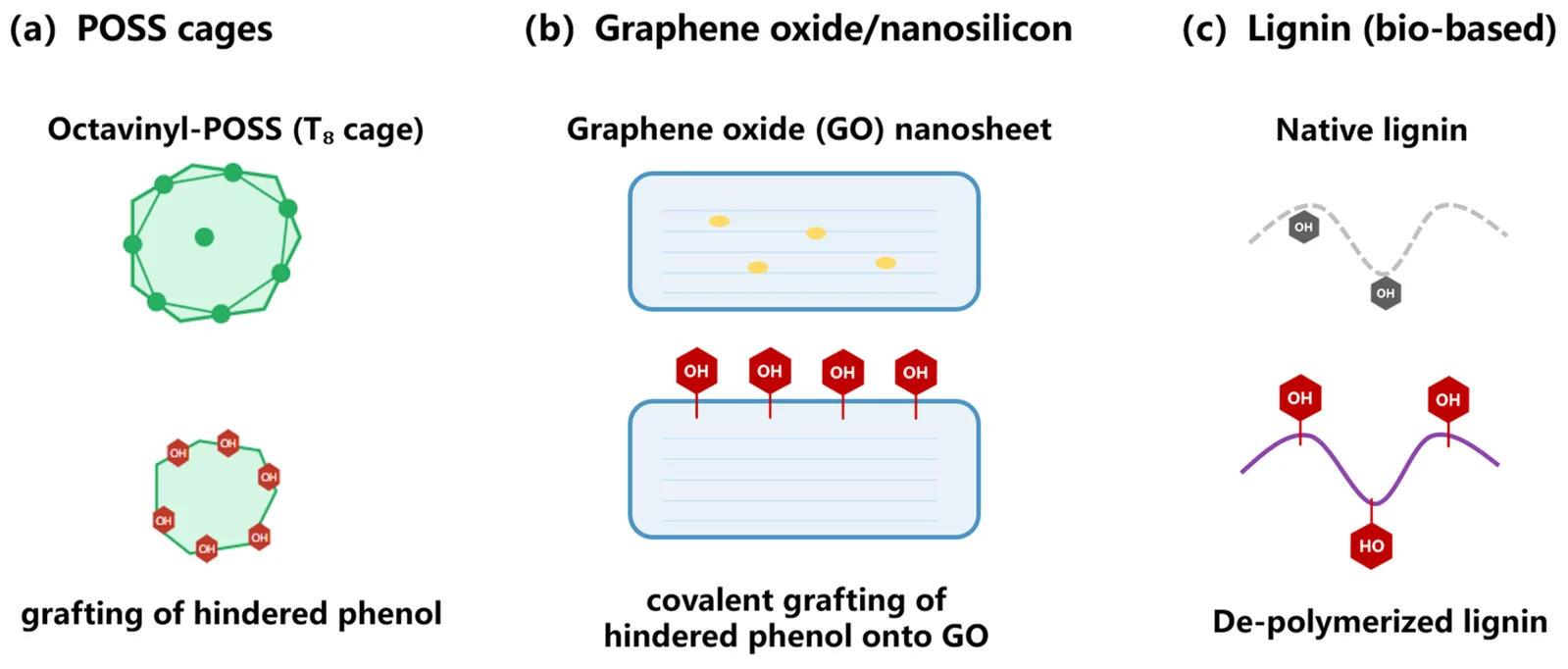
Hybrid and bio-based macromolecular phenols (Section 4.4): (**a**) hindered-phenol-grafted POSS cages; (**b**) phenol-grafted graphene oxide; (**c**) de-polymerized technical lignin.

A fourth family immobilizes hindered phenols on, or derives them from, high-molar-mass scaffolds of inorganic or biological origin (Figure 11). Inorganic carriers provide a direct route to size-based retention: a co-condensed nanosilica bearing covalently bound hindered phenol (SN-NH2-AO) has been evaluated in polypropylene [49]. The composite showed an oxidation induction time of 17 min at 180 °C, and after 3 h of extraction in dichloromethane it still retained 14.0 min (above 80% of the initial value), whereas the equivalent free antioxidant dropped sharply from 21 min to 5.4 min (only about 26% remaining), direct evidence of the anti-migration advantage of the bound form. Polyhedral oligomeric silsesquioxane (POSS) cores carrying hindered phenols have been evaluated as organic–inorganic hybrid antioxidants in polypropylene [46] and, in an amide-linked variant, in polyamide 6,6, where the rigid POSS cage combines very low volatility with defined functionality [47]: in accelerated aging at 150 °C, the growth of the carbonyl absorbance of PA66 after 35 days was cut from 0.40 (unstabilized) to about 0.10, and after 45 days the HPAm-POSS compounds still showed tensile strengths of 16–30 MPa depending on loading (against 42 MPa for the Irganox 1098 reference, i.e., substantially lower dry-oven-aging strength than the reference even at an equal phenolic-group content of 9.4 µmol g^−1^), while retaining their thermo-oxidative stability after extraction with ethanol, toluene, or ethylene glycol: conditions that physically removed Irganox 1098 and left its compound vulnerable to oxidation. Graphene oxide grafted with hindered phenol has been incorporated into polyamide 6, where the intercalated nanostructure and the grafted phenol act synergistically in thermal–oxidative stabilization [48].

Elastomers present the most demanding environment for additive retention: high chain mobility well above the glass transition, large free volume, and service conditions that combine heat, oxygen, dynamic strain, and contact with extractive media. Functionalized graphene bearing grafted hindered phenols (FGE, Figure 10) illustrates how a high-molar-mass hybrid carrier performs in natural rubber [43]. The oxidation induction time of NR vulcanizates at 170 °C rose from about 26 min for the neat rubber to 57 min with only 1 phr of FGE, essentially matching the 59 min given by 1 phr of the free phenolic antioxidant GM, although the grafted phenol groups carried by 1 phr of FGE amount to only about 0.31 phr; with 2 phr of FGE the oxidation induction time increased further to 78 min. NR/FGE vulcanizates also retained their tensile strength and elongation at break markedly better than the free-phenol control during accelerated aging at 100 °C. Because few-layer graphene can itself act as a radical scavenger and an oxygen barrier, the contributions of the grafted phenol and of the carrier cannot be separated in these data; the same caveat applies wherever the carrier is not a passive spacer.

The POSS–polypropylene study [46] repays quantitative reading because it shows both the promise and the synthetic reality of cage-type scaffolds. The synthesis, Friedel–Crafts grafting of a hindered phenol onto octavinyl-POSS, does not yield a single defined cage but a cage-rearrangement mixture of T8, T10, and T12 structures in a ratio of approximately 24:60:16. “POSS antioxidant” here therefore means a distribution of cage sizes carrying on average eight to twelve phenolic arms. This structural heterogeneity is typical of POSS derivatization chemistry and is rarely acknowledged as openly as in this work. The performance payoff, measured in polypropylene, is nonetheless substantial. The oxidation induction time rose from 38.1 min for the unstabilized reference to 50.7 min at equivalent stabilization, an increase of about one third, and anti-extraction testing against the commercial tetrafunctional standard Irganox 1010 confirmed the retention advantage expected from the cage-borne phenols. The amide-linked POSS variant in polyamide 6,6 [47] extends the concept to a polar engineering plastic, exploiting the same combination of negligible volatility and defined multi-phenol functionality. The graphene oxide study in polyamide 6 [48] adds a complementary observation. Relative to both the free-phenol control (41 min) and unmodified graphene oxide (57 min), the phenol-grafted oxide raised the oxidation induction time at 270 °C to 62 min, double that of pure PA6 (32 min), and raised the thermal degradation temperature and the activation energy of thermal decomposition simultaneously, while better preserving intrinsic viscosity and tensile strength after aging. This suggests that the grafted phenol and the nanoscale barrier act on different steps of the oxidation process, and it is a reminder that hybrid antioxidants must be benchmarked against the scaffold alone, not only against the free phenol.

Lignin, the most abundant natural polyphenol, is the bio-based counterpart, and the work of Kabir, Yuan, Kuboki, and Xu [50] provides the quantitative anchor for this branch. Their central hypothesis was that the antioxidant effectiveness of industrial lignins is limited less by chemistry than by physical form. Native technical lignins have high molar mass, few accessible phenolic hydroxyls per unit mass, and poor compatibility with nonpolar polyolefins. Catalytic de-polymerization reverses all three limitations at once: it reduces molar mass, increases the phenolic hydroxyl content, and lowers the aliphatic hydroxyl content responsible for lignin’s polarity, improving hydrophobicity and hence dispersion in polyethylene and polypropylene. At a loading of 5 wt%, the de-polymerized kraft lignin raised the oxidation induction time of polyethylene from 2.0 to 56 min and of polypropylene from 1.8 to 33 min, far above the unmodified kraft and hydrolysis lignins (6–22 min), demonstrating that a pretreatment step can convert an abundant industrial byproduct into a functional macromolecular phenol. The corollary, equally relevant to practitioners, is that lignin’s phenolic hydroxyl content is a specification that must be engineered and measured, not a constant of nature, because different feedstocks and de-polymerization processes yield materially different stabilizers. The development of lignin-based antioxidants for polymers has been reviewed [51], the property–structure relationships that govern lignin antioxidant activity, including phenolic hydroxyl content, molecular weight, and methoxy substitution, have been systematically analyzed [52], and molecular engineering of lignin has recently produced effective anti-thermo-oxidative agents specifically for natural rubber, bringing the bio-based route into the elastomer field [53]. This advantage, however, comes with an inherent trade-off that the retention framework of this review makes explicit: depolymerization lowers the molar mass of lignin, and lower molar mass means higher mobility, higher extractability, and hence potentially higher migration. The study cited above demonstrates improved oxidation induction times, but does not report migration or extraction measurements, so it cannot establish whether the retained-in-polymer fraction improves or deteriorates upon depolymerization. Until such direct retention data exist, de-polymerized lignin is more accurately classified as a bio-based active additive than as an anti-migration antioxidant.

Assessment: Hybrid and nanostructured phenols achieve very high effective molar mass per phenol and can add secondary functions (reinforcement, barrier, char formation), but they import new failure modes (dispersion and interfacial adhesion, trace-metal-catalyzed oxidation from incompletely purified bio-feedstocks, and strong intrinsic color) that conventional phenols do not have. Bio-based phenols further suffer from ill-defined and batch-variable structures, making the phenolic hydroxyl content a range rather than a specification, as the lignin case above shows concretely. These materials are scientifically active and sustainability-aligned, but quantitative, long-term benchmarking against commercial phenols under standardized conditions is still largely missing.

### 4.5. Critical Assessment and Cross-Architecture Comparison

Table 1 summarizes the four architecture families against the four evaluation criteria introduced at the start of this section (structural control, phenol content and retained activity, compatibility and dispersion, and scalability), and Table 2 collects the quantitative highlights discussed in Section 4.1, Section 4.2, Section 4.3 and Section 4.4 so that the strongest within-study results of each family can be inspected side by side. Three cross-cutting conclusions emerge from the quantitative studies discussed above. First, there is a fundamental trade-off between structural control and scalability. Copolymerization and dendrimer chemistry deliver defined molecules at costs and complexities far from industrial additive practice, whereas reactive grafting is industrially straightforward but structurally ill-defined, and its products are typically mixtures requiring extraction-based verification of the bound fraction. Hyperbranched one-pot syntheses currently offer, in qualitative terms, the most credible compromise—a judgment about structural control versus scalability, not a performance ranking—with documented 64–77% functionalization at PDI 1.3–1.7. Second, retained intrinsic activity is rarely the limiting factor. Nearly all reported macromolecular phenols outperform their monomeric analogs in retention-limited tests (although the published literature may overrepresent positive results, and direct head-to-head comparisons under sector-relevant conditions remain rare), and the elastomer case shows the carrier backbone can itself contribute synergistic stabilization. Very few studies, however, quantify phenol utilization efficiency, that is, what fraction of the immobilized hydroxyls actually participate in radical trapping inside the solid host. Third, and most important for future work, the extraction test emerges from this review as the single most informative and most underused experiment. The strongest papers in every architecture family are precisely those that quantify functional retention by comparing performance before and after extraction, ideally paired with the structural verification of covalent binding discussed in Section 3.2, yet this protocol is absent from the majority of published studies. Standardized cross-study comparison, reporting extraction fraction, OIT after extraction, and oven aging beyond roughly 1000 h together, would convert the current collection of isolated demonstrations into an engineering knowledge base, and defines the most valuable near-term contributions along with techno-economic assessment of the hyperbranched and grafted routes at pilot scale.

A methodological caution governs every quantitative comparison in this subsection. Oxidation induction times obtained at different temperatures—170 to 270 °C across the studies reviewed here—cannot be ranked directly, because a given absolute OIT increment at a lower temperature represents a physically larger improvement than the same increment at a higher temperature; likewise, equal additive mass is not equivalent to equal phenolic-hydroxyl content. Where the original studies report phenol content, we therefore discuss performance relative to phenolic functionality—for example, FGE matches the OIT of a free phenol at roughly one third of its phenol loading (Section 4.4), and the transamidation-bound phenol in PA66 matches Irganox 1098 at lower bound-phenol content (5.97 versus 8.30 µmol g^−1^, Section 5.5)—and we encourage such normalization as a field standard (Section 6.1). The comparisons above also distinguish, wherever the data permit, between improvements arising from increased functional-group content and those arising from enhanced retention. All architecture-level rankings in this review are accordingly qualitative and rest on within-study comparisons only.

## 5. Application Performance by Sector

### 5.1. Polyolefin Packaging and Films

Packaging is the sector where migration regulation makes polymer-bound phenols most immediately attractive. Conventional stabilized polyethylene films show measurable stabilizer loss and thermal-stability degradation on storage and contact [86], and the migration evidence reviewed in Section 2.1 defines the problem quantitatively: in commercial packages the standard phenolics Irganox 1010 and 1076 are present together with their phosphite synergist and its transformation products, and all migrate measurably into simulants [10]. Polymer-bound and anti-migration phenols directly address both failure modes, package protection and food contamination, and are the explicit subject of a recent review on anti-migration polypropylene antioxidants [20]. The macromolecular case studies reviewed in Section 4 translate directly into packaging-relevant evidence. The dendritic-polyethylene-bound phenol of Section 4.2 kept its oxidation induction time essentially unchanged after 15 days in boiling chloroform, conditions reported to strip BHA, Irganox 1076, and Irganox 1010 from polyethylene almost completely within 5 days, and in a direct packaging-appearance test it remained white through 21 days of oven aging in heptane while the Irganox-1010-stabilized control yellowed markedly from migrating quinoid products [30]. Hyperbranched poly(ethyleneimine)-bound phenols showed little detectable loss from polypropylene films after 70 h in n-hexane and none in water at 65 °C over 27 h, under conditions that removed Irganox 1010 almost quantitatively [83]. Co-condensed nanosilica-bound phenol in polypropylene retained more than 80% of its oxidation induction time after 3 h of dichloromethane extraction, under conditions that cut the oxidation induction time of the equivalent free antioxidant to about one quarter [49]. These are three independent demonstrations, in three architectures, of strong extractability suppression under aggressive solvent conditions. It should be noted that boiling chloroform, n-hexane, and dichloromethane tests are accelerated screening protocols, not regulatory migration tests with food simulants; demonstration of food-contact compliance would require migration data under the conditions of EU Regulation 10/2011 or equivalent frameworks. Nor does covalent immobilization automatically guarantee food-contact safety: residual unreacted monomers, low-molar-mass byproducts, degradation products, and other non-intentionally added substances may still migrate and must be assessed separately. Hybrid systems in which antioxidants are carried on mesoporous silica particles have been shown to improve thermal protection in recycled polyolefins, pointing to a practical intermediate between monomeric and fully covalent designs [60].

### 5.2. Pipes, Geosynthetics, and Infrastructure

For pressurized water pipes and geosynthetics, service lives of 50–100 years make stabilizer depletion the design-limiting factor. The depletion models for polypropylene pipes [16] and the sacrificial-versus-regenerative stabilization analysis for geosynthetics [17] together define the performance target: an antioxidant whose loss kinetics are slower than the required service time by a comfortable margin. Covalently bound phenols are conceptually ideal here, and the peroxide-crosslinked-polyethylene pipe study discussed in Section 4.1 and Section 5.3 supplies the most pipe-relevant dataset available: graftable hindered phenol/HAS combinations were retained in PEXa pipes at levels above 85% after stringent dichloromethane extraction, distributed uniformly over pipe lengths of up to 240 m, and in hydrostatic pressure testing at 115 °C and 2.5 MPa the reactively stabilized pipes outlasted the conventional Irganox 1076/HAS formulation, which failed in just over one year by oxidative embrittlement [25]. Pipe-scale data of this kind nonetheless remain rare relative to the size of the infrastructure market, and nothing comparable has been published for non-crosslinked polyolefin pressure pipes. Hindered phenolic antioxidants remain the standard heat-oxygen stabilizers in high-density polyethylene (HDPE) for such durable goods, providing the baseline against which macromolecular candidates must be benchmarked [87].

### 5.3. Electrical and Electronic Applications

Two electrical niches illustrate where migration control is worth a significant cost premium. In metallized polymer film capacitors, additive migration and volatilization degrade the dielectric interface; functional polypropylene containing covalently bound hindered phenols was developed specifically for this application and represents one of the few copolymer-route systems driven by an actual device requirement [28]. In peroxide-crosslinked polyethylene cable insulation, the antioxidant must survive the crosslinking step without interfering with it or being extracted in service; reactive antioxidants grafted during peroxide crosslinking address exactly this constraint [25], with retention above 85% after solvent extraction and a lower interference with peroxide cure than the conventional Irganox 1076 control, as quantified in Section 4.1 and Section 5.2.

### 5.4. Elastomers

Natural rubber and related elastomers combine high chain mobility with extractive service environments, and are therefore among the earliest beneficiaries of macromolecular phenol design [43,44,45]; the quantitative data discussed in Section 4.4 (an oxidation induction time matching that of the free phenolic antioxidant at only about one third of its phenol content, and improved retention of tensile properties after aging at 100 °C) illustrate the magnitude of benefit achievable in this sector. Dendritic amine-bearing antioxidants have been demonstrated in acrylonitrile–butadiene rubber [40], and molecularly engineered lignin has recently been shown to function as an effective anti-thermo-oxidative agent for natural rubber, opening a bio-based route for the tire and rubber-goods sector [53]. The open questions for this sector are the influence of the macromolecular additive on vulcanization chemistry and dynamic mechanical properties, which current studies report only partially.

### 5.5. Engineering Plastics

Engineering plastics offer a binding opportunity that polyolefins lack: a reactive backbone. Polyamides are the clearest case, because transamidation during melt processing allows reactive phenols to be grafted into the host in situ. Hindered phenols bonded through para bridge groups of different structure have been compared systematically in polyamide 6 [84]. The variant bridged through an aromatic ring (HPAP) showed the best radical-scavenging activity and the best migration resistance in PA6: after 12 days of thermal–oxidative aging at 150 °C, PA6/HPAP retained 117% of its tensile strength and 68% of its notched impact strength relative to the unaged sample, a performance comparable to that of PA6 stabilized with the commercial Irganox 1098. The retention above 100% reflects the initial aging-induced increase in tensile strength reported in that study, plausibly arising from annealing or further crystallization at 150 °C, rather than a strengthening effect of the antioxidant, and the figure is quoted here as reported. A hindered phenolic antioxidant engineered for high transamidation reactivity provides long-term thermo-oxidative stabilization of polyamide 6,6 [85]. Computational design, targeting an amide resonance energy of only 6–7 kcal mol^−1^ on a triazine ring, enabled about 60% grafting during ordinary twin-screw extrusion, and the grafted sample carrying 5.97 µmol g^−1^ of bound phenol matched the accelerated-aging performance of 8.30 µmol g^−1^ of Irganox 1098. That is comparable stabilization with about 28% less phenolic active substance. The authors attribute this efficiency to grafting, which suppresses physical loss; because the bound phenol differs from Irganox 1098 in molecular structure as well as in mobility, a contribution from different intrinsic radical-scavenging activity cannot be excluded, and the comparison is best read as a first-order one within a single host and test protocol. It is nonetheless a notable example of designing the anchoring chemistry to match the host’s native reactivity rather than importing a generic grafting reaction. POSS-based hindered phenols [47] and graphene-oxide-grafted phenols [48] extend the toolbox with hybrid nanostructures for polyamides, with quantitative records that bear directly on coolant-contact and under-hood service. The amide-linked POSS phenol kept PA66 oxidatively stable after extraction with ethanol, toluene, or ethylene glycol, conditions that stripped Irganox 1098 and left its compound vulnerable, and cut the carbonyl-absorbance growth after 35 days at 150 °C from 0.40 to about 0.10 [47], while the phenol-grafted graphene oxide doubled the oxidation induction time of PA6 at 270 °C (32 to 62 min), outperforming both the free phenol (41 min) and graphene oxide alone (57 min) [48]. The importance of retention in these hosts is underlined by aging studies that track the actual concentration of hindered phenols in polyamide 11 during thermal aging, showing progressive phenol depletion as the life-limiting quantity [69], and by fracture studies showing that diffusion-limited oxidation of unstabilized polyamide 6 proceeds through three distinct stages of surface-induced embrittlement [88].

### 5.6. Biomedical Polymers

This section steps outside the scope of macromolecular phenols to examine the most commercially successful retention-first phenol strategy in any polymer sector: α-tocopherol (vitamin E) stabilization of ultra-high-molecular-weight polyethylene (UHMWPE) for joint implants [55,56]. Vitamin E is a monomeric hindered phenol (molar mass ≈ 430 g mol^−1^), not a polymer-bound antioxidant. It is discussed here as a benchmark and as an instructive case of diffusion-based incorporation with partial irradiation-induced grafting, because it demonstrates what the market rewards when retention is engineered properly, and what quantitative targets a polymer-bound phenol must meet to displace it. The boundary conditions of this application are instructive for any developer of polymer-bound phenols. Because vitamin E scavenges the very radicals needed for radiation crosslinking, the blend route is limited to concentrations below about 0.3 wt%; above this level, crosslink density cannot be raised to target values even at irradiation doses as high as 200 kGy. Yet remarkably little phenol is needed for stabilization. Blends containing as little as 125 ppm of vitamin E, irradiated up to 75 kGy, passed two weeks of accelerated aging at 70 °C under 5 atm oxygen (ASTM F2003) without measurable oxidation, and vitamin E-diffused, irradiated UHMWPE showed about 58% higher fatigue strength than irradiated-and-melted material of the same dose, because the post-irradiation melting step that destroys crystallinity could be avoided. Two further observations connect this benchmark directly to the themes of the present review. First, a fraction of the vitamin E has been reported to become grafted onto the polyethylene chains during terminal sterilization irradiation, which may reduce elution; the grafted fraction and the residual activity of the grafted species, however, remain poorly quantified, so the extent of covalent anchoring achieved in practice is uncertain. It should be noted, however, that the primary stabilization mechanism remains the intrinsic radical-scavenging activity of the dissolved tocopherol, and quantitative data on the grafted fraction and the residual activity of the grafted species are still limited in the open literature. Second, the entire clinical rationale is retention: the phenol must remain active in the device for decades in vivo, which is precisely the requirement that monomeric phenols fail in less demanding applications. The behavior of natural phenolic antioxidants in polyolefins and other polymer matrices has been characterized in detail, including their activity and fate during melt processing and photooxidation [89,90,91], and novel natural-based antioxidants continue to be developed for the thermal stabilization of polyethylene [92] (ref. [92] describes a non-phenolic, natural-based phosphine antioxidant and is cited here only for completeness, as an example of stabilization chemistry beyond the phenolic class.) For the polymer-bound phenol field, the lesson and the open target are the same: a covalently bound, biocompatible hindered phenol that reproduces the ppm-level efficiency and decades-long retention of vitamin E in implant-grade UHMWPE would have a clear clinical and commercial value proposition.

### 5.7. Recycling and the Circular Economy

**Figure 12:**
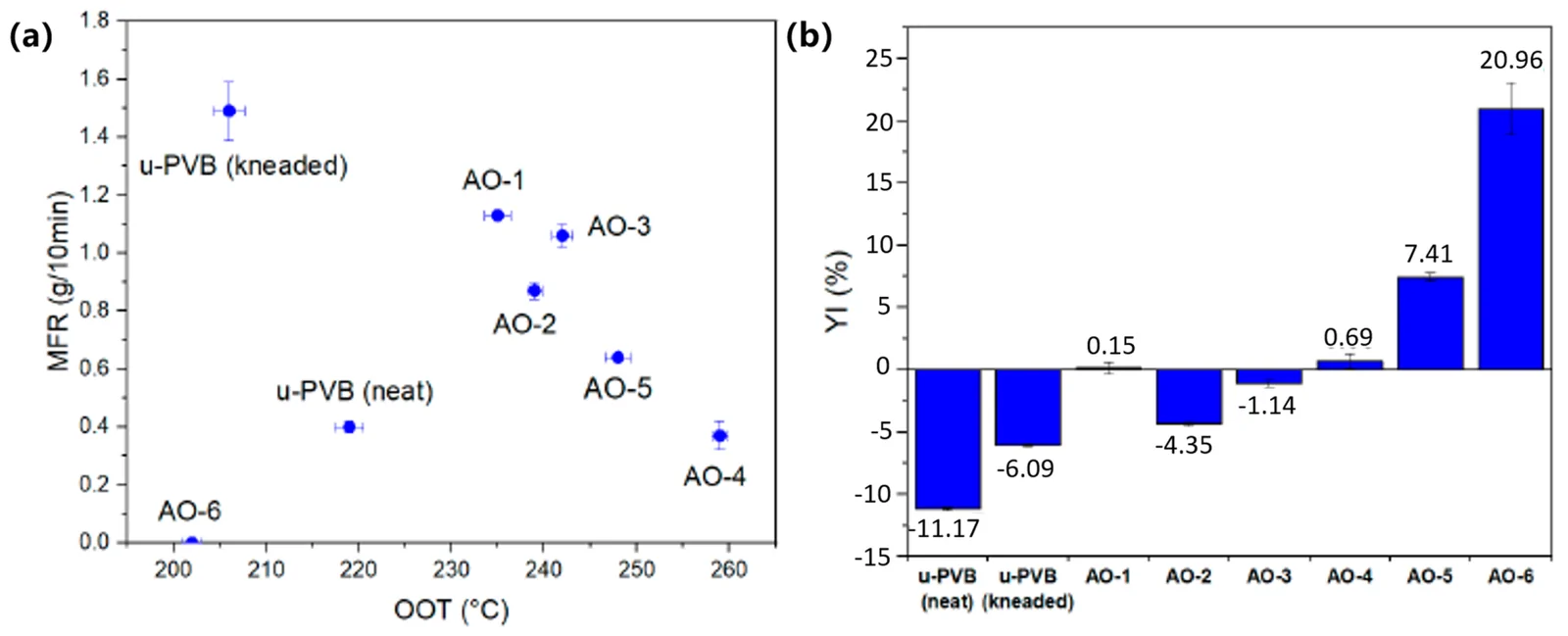
Screening of antioxidant formulations (AO-1 to AO-6) for closed-loop recycling of poly(vinyl butyral) film: (**a**) oxidation onset temperature versus melt flow rate; (**b**) yellowness index change [62]. Reproduced from ref. [62] under the terms of the CC BY 4.0 license.

**Figure 13:**
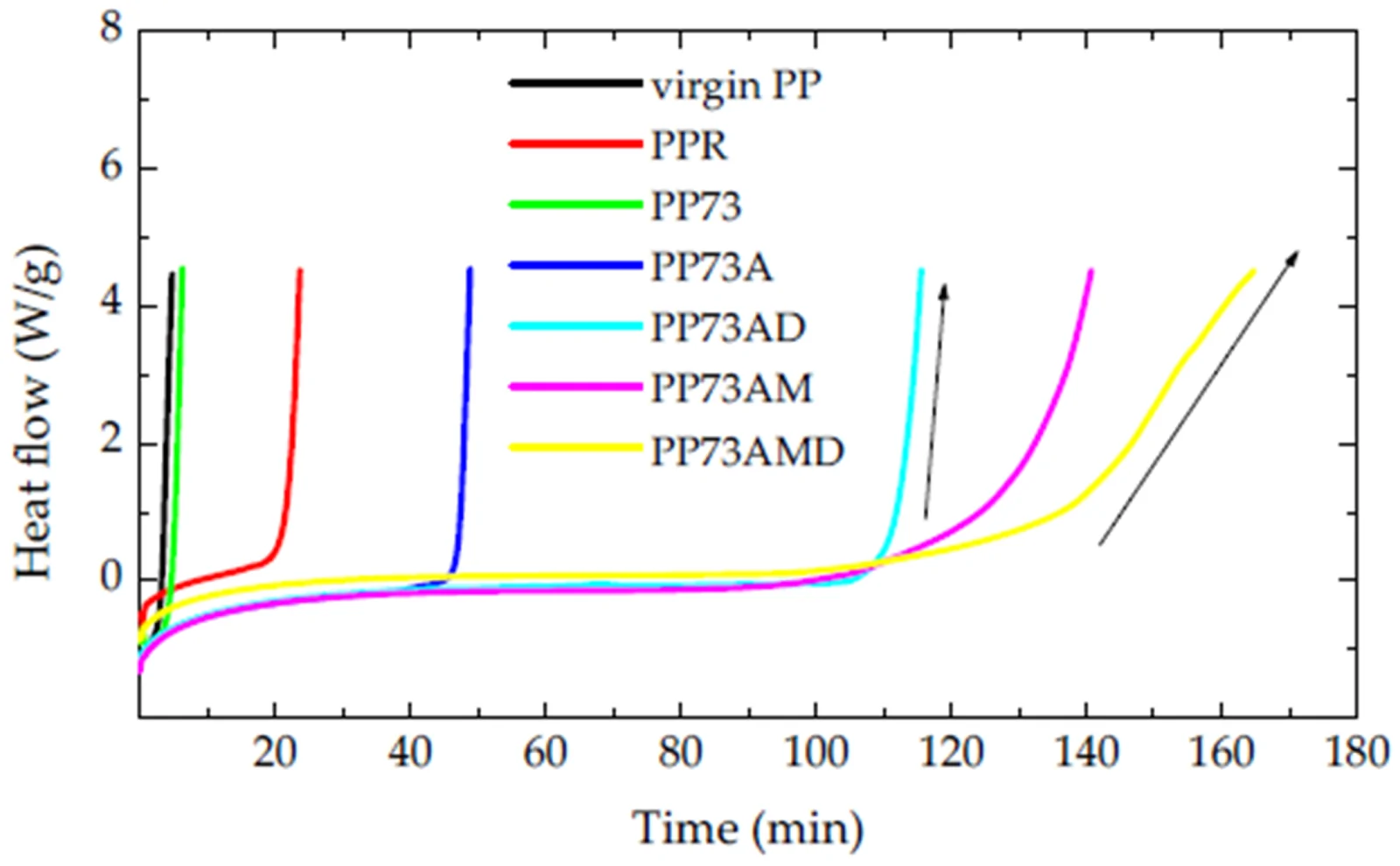
Isothermal oxidation induction time curves at 180 °C for virgin PP, recycled PP, and 70:30 virgin/recycled blends restabilized with Irganox 1076 in free form or carried on mesoporous MCM-41 silica particles [60]. Adapted from ref. [60] under the terms of the CC BY 4.0 license.

Mechanical recycling concentrates the physical-loss problem: each reprocessing cycle consumes stabilizer chemically while heat and shear accelerate volatilization, so recyclates arrive under-stabilized. Restabilization is therefore mandatory, and its effectiveness can be monitored with sensitive oxidation methods such as chemiluminescence, as demonstrated for recycled polypropylene [57]. Studies on reprocessed acrylonitrile–butadiene–styrene (ABS) from waste electrical equipment [58] and on metallocene polypropylene [59] confirm that antioxidant choice during recycling strongly affects retained properties. A closed-loop study on poly(vinyl butyral) interlayer film screened six restabilization formulations by oxidation onset temperature and yellowness-index change [62] (Figure 12). Within this context, low-volatility and polymer-bound phenols lose less activity per cycle, and hybrid silica–antioxidant particles improve thermal protection in recycled polyolefins [60] (Figure 13). These MCM-41-supported systems retain the antioxidant by adsorption and confinement rather than by macromolecular bonding; they are included because recycling service is precisely where the practical boundary between carrier-bound and polymer-bound retention is decided.

The restabilization of post-consumer recycled polypropylene with natural antioxidants extracted from spent coffee residue [61] provides a quantitative case study of what a stabilization package must survive in recycling service. The extract is a structurally undefined mixture of natural phenolics, physically blended rather than bound; it is discussed here because recycling is the sector in which such low-cost bio-based additives are most likely to see near-term adoption, and because its multi-cycle protocol is methodologically exemplary for retention testing. The extract, added at 1.0 wt%, was subjected together with the recyclate to five consecutive extrusion cycles. In the unstabilized recyclate, reprocessing produced the classic degradation signature, increasing melt flow rate from chain scission together with falling oxidation induction time and onset temperature, whereas the extract-stabilized material maintained stable melt-flow rate (MFR) and higher OIT and onset values across all five cycles, matching the behavior of a commercial synthetic primary antioxidant run in parallel. Two implications stand out. First, five melt passes constitute a far more demanding test than any single processing step; the stable MFR and OIT behavior observed is consistent with antioxidant retention, although it does not directly demonstrate it, because an additive that is consumed or transformed during processing could in principle leave the same apparent processing signature. Multi-cycle protocols of this kind should nevertheless become standard for anti-migration claims, ideally paired with direct retention measurements. Second, the parity achieved by an unoptimized natural extract against a commercial phenol is compatible with the view that the performance bar in recycling applications is set by retention and dispersion rather than by intrinsic phenol activity—the regime in which macromolecular designs should excel—but it is equally compatible with the alternative that the extract combines higher intrinsic activity with poorer retention; distinguishing the two explanations requires direct retention measurements such as post-extraction OIT or chemical analysis of the aged compound. Closed-loop recycling of poly(vinyl butyral) interlayer film via restabilization illustrates the same principle in a specialty stream [62]. At the strategy level, recent reviews frame additive design for durability and circularity as a single coupled problem [63], with natural-antioxidant routes for bio-based and biodegradable plastics forming a complementary branch [64] and clay-supported stabilizer systems extending retention concepts to nanocomposites [65]. For macromolecular hindered phenols, recycling is arguably the fastest-growing opportunity: the value proposition, surviving multiple heat histories without migrating, aligns exactly with what recyclate quality demands.

### 5.8. Cross-Sector Assessment

The sector-level mapping of stability issues, retention requirements, and suitable architectures is summarized in Table 3. Across sectors, adoption readiness varies inversely with the tolerance for additive cost and regulatory scrutiny. Biomedical UHMWPE shows that a retention-first phenol can dominate a high-value market, and it sets concrete quantitative benchmarks: effective stabilization at hundreds of ppm, and decades-long in vivo retention. Electrical applications show that covalent architectures are already justified where migration destroys device function. Packaging and infrastructure, the largest-volume opportunities, await quantitative long-term data and cost-competitive scalable syntheses. Recycling may provide the near-term volume driver, because restabilization is already standard practice into which superior retention can be dropped. For developers of polymer-bound hindered phenols, the practical implication is clear: prioritize application-specific validation, meaning extraction plus performance under sector-relevant, multi-cycle protocols, over further architectural novelty.

## 6. Challenges and Outlook

### 6.1. Standardization of Anti-Migration Verification

The most immediately actionable improvement identified in this review is methodological. Across all four architecture families, the most convincing papers share one experiment: a performance measurement paired with a retention measurement, typically a comparison of oxidation induction time or aging resistance before and after solvent extraction [21,29,39]. Yet this protocol appears in only a minority of published studies, and where it appears, extractants, severities, and reported quantities differ. A minimal reporting standard (extractable fraction under a polar and a nonpolar extractant, OIT before and after extraction, and a defined oven-aging endpoint) would make anti-migration claims comparable across laboratories and would immediately raise the evidentiary quality of the field.

### 6.2. Phenol Utilization Efficiency in the Solid State

Nearly all macromolecular phenols outperform monomeric analogs in retention-limited tests (although the published literature may overrepresent positive results), but almost nothing is known about what fraction of the immobilized phenolic hydroxyls actually participates in radical trapping inside a solid, semi-crystalline host. Phenols buried in domains inaccessible to peroxy radicals contribute molar mass but not activity. Quantifying utilization efficiency, for example by combining phenol-content assays with post-aging residual-activity measurements, would convert architecture design from an empirical to a rational exercise, and would clarify the true trade-off between phenol density per molecule and accessibility.

### 6.3. Long-Term and Application-Scale Validation

The gap between laboratory demonstration and application validation remains wide. Oven aging beyond roughly 1000 h, extraction in real service fluids, multi-cycle reprocessing, and field trials are reported in only a small minority of the studies reviewed. The sectors with the clearest quantitative targets (pipes and geosynthetics with 50–100-year design lives [16,17], and implant-grade UHMWPE with decades-long in vivo retention [55]) are precisely those where macromolecular phenols have the least published long-term data. Application-scale validation, not further architectural novelty, is the current bottleneck.

### 6.4. Scalable Synthesis and Techno-Economics

The architecture comparison of Section 4.5 identifies a persistent trade-off between structural control and scalability. Reactive grafting is continuous, solvent-free, and uses standard equipment, but yields ill-defined mixtures; copolymerization gives defined structures at specialty-polymerization cost; one-pot hyperbranched synthesis currently offers the best documented compromise between functionality, reproducibility, and process simplicity [39]—a qualitative judgment based on within-study evidence, given the protocol differences discussed in Section 4.5. Techno-economic assessment at pilot scale (including the cost of monomer purification, initiator consumption, and downstream removal of unreacted phenol) has not been published for any of these routes and is a prerequisite for industrial adoption.

Cost considerations reinforce this hierarchy. We emphasize that the cost comparisons in this subsection are a qualitative assessment based on publicly discernible process features—number of synthetic steps, raw-material availability, and compatibility with existing industrial equipment—rather than on published cost data; to our knowledge, no pilot-scale techno-economic assessment has been published for any of the routes reviewed here, and this gap itself constitutes an open research need (Section 6.4). The linear oligomeric phenols of Section 4.3.1 are produced by established condensation chemistry at commodity scale from bulk feedstocks, and on this basis their per-kilogram cost can be expected to be of the same order as that of Irganox-type monomeric phenols, which is consistent with their decades-long dominance in high-volume rubber and latex applications. Reactive grafting adds little more than the price of the functional monomer, the initiator, and a devolatilization step to an extrusion line that already exists, giving it the lowest market-entry barrier of the covalent routes. Copolymerization routes and dendritic or POSS-based architectures occupy the opposite end: multi-step syntheses, purification by precipitation, and specialty catalysts raise the cost per kilogram of bound phenol well above commodity levels, so their adoption is realistic only where retention commands a premium, as in biomedical polymers, infrastructure, or engineering resins. A practical implication for high-volume applications is that architectures should be compared on the basis of cost per year of stabilized service rather than cost per kilogram of additive, a metric that favors high-retention phenols whenever restabilization or premature product replacement is expensive.

### 6.5. Bio-Based Feedstocks and the Circularity Driver

Bio-based macromolecular phenols (de-polymerized lignins and engineered natural polyphenols) are advancing rapidly [50,51,52,53]. The stabilization study of Nanni et al. on poly(butylene succinate) (PBS) [64] illustrates how decisively the polyphenol composition of a natural extract governs its performance: a commercial grape-seed extract containing 55% total polyphenols, dominated by polymeric proanthocyanidins, preserved the molecular weight of PBS over six consecutive processing cycles and nearly 300 h of oven aging at 110 °C, performing comparably to Irganox 1010, whereas a lab-prepared grape-pomace extract containing only 15.9% total polyphenols, mainly of oligomeric character, was markedly less effective and even accelerated PBS degradation, because its catecholic polyphenols auto-oxidize to peroxide and radical species under aging conditions [64]. Neither extract is a structurally defined macromolecular phenol—both are physically incorporated mixtures—but the study is instructive precisely because it isolates polyphenol content and degree of polymerization as the variables that decide whether a natural extract stabilizes or degrades its host. This contrast underlines that natural phenolic extracts are not automatically anti-migration stabilizers: a high content of polymeric polyphenols favors both activity and retention, whereas oligomeric, readily oxidized fractions can become pro-oxidant. These bio-based systems are driven both by sustainability goals and by the economics of recycling, where restabilization is already standard practice and retention across multiple heat histories is the decisive property [57,60,61]. The open problems are reproducibility (phenolic hydroxyl content as a controlled specification rather than a batch variable), color, and trace-metal purity. If these are solved, bio-based macromolecular phenols could become the volume driver that brings the whole class into commodity applications.

## 7. Conclusions

This review has treated the macromolecular hindered phenol field as an engineering problem rather than a catalog of syntheses. In the retention-limited test protocols that dominate the literature reviewed here, the decisive variable is whether the phenol stays where it is needed: under such conditions, macromolecular and polymer-bound architectures consistently outperform their monomeric counterparts. This does not mean that radical-scavenging chemistry has ceased to matter—intrinsic activity, phenolic accessibility, phenol regeneration, and transformation chemistry still govern performance once retention is secured, and under conditions where physical loss is slow, commercial monomeric phenols can match or outperform macromolecular systems.

Several conclusions hold across the literature reviewed here. First, covalent binding and high molar mass both suppress physical loss. Covalent binding offers the most robust retention under aggressive extraction, although confinement-based and low-solubility high-molar-mass systems can also survive specific extraction protocols; distinguishing covalent from non-covalent retention requires structural characterization, because extraction data alone demonstrate functional retention rather than the chemistry of attachment. Second, structural control and scalability remain in tension. Copolymerization and dendrimer chemistry give defined molecules, while reactive grafting gives industrial simplicity, and hyperbranched one-pot syntheses currently offer the most credible compromise between the two. Third, the performance bar in most applications is set by retention and dispersion rather than by intrinsic phenol activity. This is consistent with both the clinical adoption of vitamin E-stabilized UHMWPE—in which retention operates alongside crosslinking, residual-radical control, crystallinity, fatigue, wear, sterilization, and processing history—and the parity of unoptimized natural extracts with commercial phenols in recycling service.

The path forward is methodological and molecular in equal measure. Methodological harmonization is the most urgent need, but molecular challenges remain open: linker stability, phenolic accessibility, compatibility, color, toxicity, recyclability, and phenol utilization efficiency all require molecular solutions. Community adoption of a minimal anti-migration reporting standard, cross-laboratory comparison under identical protocols, quantification of phenol utilization efficiency, and pilot-scale techno-economic assessment would convert two decades of inventive synthesis into a stabilization technology base. Until then, the strongest contribution a new study can make is not another architecture, but another verified one.

## Figures and Tables

**Table 1:** Qualitative comparison of macromolecular hindered-phenol architectures against the four evaluation criteria: structural control, phenol content and retained activity, compatibility and dispersion, and scalability.

Architecture Family	Structural Control	Phenol Content/Activity	Compatibility & Dispersion	Scalability
Linear oligomeric phenols (e.g., p-cresol–DCPD resins)	Low (ill-defined oligomer mixtures)	Moderate OH density; proven industrial activity	Good in rubbers and polyolefins	High (only family with large-scale industrial adoption)
Grafted polyolefins (reactive processing)	Low (statistical graft distribution; mixture with free monomer and homopolymer)	Moderate; bounded by graft level; bound fraction must be verified by extraction	Excellent (polyolefin backbone)	High (continuous reactive extrusion; solvent-free)
Phenol-bearing copolymers	High (catalytic control of M, topology, comonomer; TGA assay for bound phenol)	Reproducible, tunable density; OIT scales with bound-phenol index	Intrinsic when backbone matches host	Low–moderate (specialty polymerization)
Dendritic/hyperbranched	High (dendrimers)/moderate (hyperbranched, PDI 1.3–1.7)	Highest phenol density per molecule; 64–77% site functionalization documented; multifunctional designs possible	Polar cores can hinder dispersion in nonpolar hosts	Medium (dendrimers: multi-step conventional synthesis; hyperbranched: one-pot, laboratory-scale only)
Hybrid, bio-based, nanostructured (POSS, GO, lignin)	Variable; ill-defined for lignin (PhOH is process-dependent)	Variable; added secondary functions; lignin effective at ~5 wt% after de-polymerization	Dispersion/interface is the key challenge	Medium at best; no industrial-scale demonstration (lignin: bulk feedstock but laboratory-scale functionalization; nanofillers: multi-step preparation)

Note: Ratings are qualitative assignments based on the representative studies cited in Section 4.1, Section 4.2, Section 4.3 and Section 4.4, not on standardized measurements. To make the assignments reproducible, fixed criteria were applied. Structural control refers to how precisely molar mass, architecture, and phenol placement can be specified and reproduced; it is rated high when the architecture is defined by the synthesis itself (composition set by stoichiometry, with dispersity and functionalization quantified) and low when the product is a statistical mixture (e.g., POSS cage-size distributions). Phenol content and retained activity is rated high where the phenolic hydroxyl content per unit mass is quantified and the retained radical-scavenging activity relative to the free phenol analog is reported, medium where only one of the two is quantified, and low where neither is reported. Compatibility and dispersion is rated high where dispersion in the intended host is demonstrated directly (e.g., microscopy, solubility, or property retention at loading) or is intrinsic to a matched-backbone design, medium where it is inferred indirectly, and low where incompatibility or aggregation is documented. Scalability is rated high when processing on continuous industrial equipment (e.g., twin-screw extrusion) has been demonstrated, medium for one-pot syntheses from commercial monomers and other multi-step but conventional syntheses, and low where organometallic polymerization or specialty catalysts are required; the dividing line is whether scale-up on existing industrial equipment has been demonstrated in the cited studies—high ratings reflect documented industrial-scale operation, whereas one-pot synthesis is treated as laboratory-scale synthetic simplicity and is not, by itself, evidence of industrial scalability.

**Table 2:** Quantitative highlights of the representative studies reviewed in Section 4.1, Section 4.2, Section 4.3 and Section 4.4, organized with one row per study so that each result can be traced to its reference (test conditions differ between studies; values are within-study comparisons against the stated controls, not cross-study benchmarks).

Study (Ref.)	Architecture Family (Subsection)	Matrix and Loading	Test/Extraction/Aging Conditions	Key Quantitative Results	Control
Wingstay L/Lowinox CPL (industrial baseline)	Linear oligomeric phenols (Section 4.3.1)	SBR, latex, polyolefins; typical use level 0.05–0.5 wt%	Decades of industrial service data	Low volatility and extraction relative to monomeric phenols at Mn only ~600–700 g/mol	- (industrial baseline)
Arabi et al. [21]	Grafted polyolefins (Section 4.1)	POE grafted with unsaturated hindered phenol esters by peroxide-initiated reactive processing	OIT comparison against physical-blend counterparts	Grafted POE reached ~10 min (redox-initiated grafting), clearly superior to the corresponding physical blends	Physical blends of the same phenols
Manteghi et al. [22]	Grafted polyolefins (Section 4.1)	3 wt% phenol-functionalized ethylene copolymer (POE or EVA carrier) in unstabilized PP powder (2000 ppm phenol)	OIT at 210 °C; ethanol extraction at 70 °C for 1–7 days	OIT 11.5 min (POE carrier) and 9.5 min (EVA carrier) vs. 1.3 min for equivalent free phenolic acid; OIT retained through ethanol extraction, while Irganox 1010/PP fell to 4.2 min after 3 days	Equivalent free phenolic acid; Irganox 1010/PP
Manteghi et al. [23]	Grafted polyolefins (Section 4.1)	Phenol covalently attached to surface-functionalized PP; PP/PP-bound-phenol blend at 3:1	Melt rheology for 100 min; successive extraction in acetone at 50 °C and hexane at 70 °C, 3 days each	No measurable viscosity decay over 100 min in the melt, retained after both extraction steps; control degraded rapidly	Conventionally stabilized PP
Latent-antioxidant grafting [24]	Grafted polyolefins (Section 4.1)	Polyolefins grafted with phenol precursor structures	Peroxide-initiated melt modification	Grafted precursors survive processing and liberate the active phenol subsequently (qualitative)	-
PEXa graftable phenol/HAS [25]	Grafted polyolefins (Section 4.1)	PEXa pipes stabilized with graftable phenol/HAS system	Stringent dichloromethane extraction; hydrostatic test at 115 °C/2.5 MPa	Retention > 85% after DCM extraction, far above the conventional formulation; graftable pipes far outlasted the conventional formulation, which failed in just over one year	Conventionally stabilized PEXa pipe (Irganox 1076/HAS)
Mechanochemical and coagent grafting [26,27]	Grafted polyolefins (Section 4.1)	BHT-type phenols grafted onto PP-g-MA; tea-polyphenol coagent at 0.5–3 wt%	Solvent-free mechanochemical grafting	Mechanochemical route requires neither solvent nor extrusion; coagent markedly raises grafting efficiency	-
Balzadeh and Arabi [29]	Phenol-bearing copolymers (Section 4.2)	Ethylene–phenol copolymers blended into PP at equal SHP groups per gram	OIT at 185–200 °C; solvent extraction	OIT tunable over ca. 1–36 min with phenolic comonomer content; post-extraction OIT slightly exceeded pre-extraction values	Unextracted copolymer; monomeric phenol controls
Balzadeh et al. [30]	Phenol-bearing copolymers (Section 4.2)	Dendritic phenolic-antioxidant-functionalized polyethylene	Boiling chloroform for 15 days; oven aging in heptane for 21 days	OIT essentially unchanged after 15 days in boiling chloroform, under conditions that stripped BHA, Irganox 1076, and Irganox 1010 almost completely within 5 days; no yellowing after 21 days	BHA; Irganox 1076/1010; Irganox-1010-stabilized PE
EVA-derived copolymer [31]	Phenol-bearing copolymers (Section 4.2)	EVA hydrolyzed and transesterified with hindered-phenol propionate; used as PP stabilizer (PP-PE)	Melt flow index over three consecutive extrusion passes	Stable MFI across three passes, outperforming Irganox 1010 and Irganox 1076 controls (Figure 8)	Irganox 1010; Irganox 1076; unstabilized PP
ADMET oligomers [34]	Phenol-bearing copolymers (Section 4.2)	ADMET phenolic oligomers (Mn about 2600 g/mol), with and without polythioether synergist	OIT at 185–200 °C	ADMET phenol: 34 min vs. 41 min for Irganox 1010 (200 °C); phenol + polythioether: 339 min at 185 °C vs. 115 min (phenol alone) and 57 min (Irgafos 168)	Irganox 1010; Irgafos 168
Dendritic generations [35]	Dendritic/hyperbranched (Section 4.3.2)	Three dendritic generations in lubricant base oil at 0.5 wt%	Pressurized-DSC screening; TGA	All generations more thermally stable than and ranked ahead of BHT, Irganox L135, and Irganox L57	BHT; Irganox L135; Irganox L57
Diamine-bridged phenols [36]	Dendritic/hyperbranched (Section 4.3.2)	PP and LLDPE at equal phenolic-hydroxyl concentration	MFR retention, OIT, oven aging	Antioxidant activity and mechanical-property retention increased with bridge length at equal phenolic-OH content	Monomeric bridged-phenol series
Dendrimer-bridged phenols [37]	Dendritic/hyperbranched (Section 4.3.2)	PP and LLDPE	OIT, aging coefficient; DPPH assay in solution	Solution (DPPH) activity decreased with bridge length, yet in-polymer OIT and aging coefficients increased	Monomeric phenols
HbPG nanoparticles [39]	Dendritic/hyperbranched (Section 4.3.2)	PVC blends (0.14–1 eq. phenol per 100 VC units)	OIT by DSC; hexane and water extraction for 240 h	OIT of unstabilized PVC (~400 s) raised by a factor of 2.5–3, close to Irganox 1010; after 240 h only 8% leached by hexane and 3% by water vs. >30% and ~9% for Irganox 1010	Irganox 1010
PEI-bound phenols [83]	Dendritic/hyperbranched (Section 4.3.2)	PP films (0.2 wt% phenol groups)	Hexane at room temperature for 70 h; water at 65 °C for 27 h	Low extraction in hexane under conditions that removed Irganox 1010 almost completely; no extraction detected in hot water for any sample, Irganox 1010 included	Irganox 1010
Star oligomer PM [44]	Dendritic/hyperbranched (Section 4.3.2)	EPDM; PM (1544 g/mol) vs. monomeric MC (264 g/mol)	Aging at 120 °C for 15 days; XPS surface analysis	XPS surface nitrogen rose only 0.55 to 2.08% (PM) vs. 0.69 to 4.25% (MC); PM raised crosslink density and reduced curing interference	Monomeric MC; PETPM+MC mixture
PHPBT-b-TPH [45]	Grafted polyolefins (Section 4.1)	NR vulcanizates, 1 phr macromolecular thiobisphenol	Thermo-oxidative aging at 100 °C for 168 h	Tensile-strength retention 43.6% vs. 35.6%, and elongation retention 58.6% vs. 54.5%, versus the corresponding low-molar-mass thiobisphenol TPH	Low-molar-mass TPH at equal loading
Nanosilica-bound phenol [49]	Hybrid/nanostructured (Section 4.4)	PP + co-condensed nanosilica-bound phenol (SN-NH2-AO)	OIT at 180 °C; dichloromethane extraction for 3 h	OIT 17 min vs. 21 min for free AO; after extraction 14.0 min retained (>80%) vs. 5.4 min (25.8%) for free AO	Equivalent free AO
AO-POSS in PP [46]	Hybrid/nanostructured (Section 4.4)	PP + AO-POSS at equimolar hindered phenol relative to Irganox 1010	OIT; Soxhlet extraction for 48 h	OIT 50.7 min (AO-POSS/Irgafos 168) vs. 38.1 min (Irganox 1010/Irgafos 168); 69.5% vs. 32.2% of OIT retained after Soxhlet extraction	Irganox 1010 (+/− Irgafos 168)
HPAm-POSS in PA66 [47]	Hybrid/nanostructured (Section 4.4)	PA66 + 0.35% amide-linked POSS phenol	Aging at 150 °C for 35–45 days; ethanol, toluene, and ethylene glycol extraction	Carbonyl growth after 35 days cut from 0.40 to ~0.10; stability retained after extraction that physically removed Irganox 1098; tensile 16–30 MPa after 45 days vs. 42 MPa for Irganox 1098	PA66/Irganox 1098
Phenol-grafted GO [48]	Hybrid/nanostructured (Section 4.4)	PA6 + 0.5 wt% GO-HP	OIT at 270 °C	OIT raised from 32 to 62 min, vs. 41 min for free phenol and 57 min for GO alone	PA6/HP; PA6/GO
De-polymerized lignin [50]	Hybrid/bio-based (Section 4.4)	PE and PP + 5 wt% de-polymerized lignin	OIT	OIT raised from 2.0 to 56 min (PE) and from 1.8 to 33 min (PP)	Neat PE/PP
Phenol-functionalized graphene FGE [43]	Hybrid/nanostructured (Section 4.4)	NR vulcanizates, 1–2 phr FGE (~0.31 phr phenol at 1 phr)	OIT at 170 °C; aging at 100 °C	OIT 26 min (neat) to 57 min (1 phr FGE), matching 59 min for 1 phr free GM; 78 min at 2 phr; better tensile and elongation retention than free GM during aging	Neat NR; free GM phenol
HPAP in PA6 [84]	Hybrid/bio-based (Section 4.4)	PA6 with aromatic para-bridged phenol grafted by transamidation	Aging at 150 °C for 12 days	117% tensile and 68% notched-impact retention, comparable to Irganox 1098	PA6/Irganox 1098
Transamidation-bound phenol in PA66 [85]	Hybrid/bio-based (Section 4.4)	PA66 with reactively grafted phenol	Grafting quantification; aging	~60% grafting; 5.97 umol/g bound phenol matched the performance of 8.30 umol/g Irganox 1098	Irganox 1098

Note: All values are taken from the original studies cited in the corresponding sections; test temperatures, loadings, and extraction media differ between studies, so the table supports within-study (bound versus free/conventional) comparisons only. Data for elastomer-targeted systems are listed under their topological families (see note to Table 1).

**Table 3:** Sector-level mapping of primary stability issues, retention requirements, and macromolecular architectures reported for each sector (details in Section 5.1, Section 5.2, Section 5.3, Section 5.4, Section 5.5, Section 5.6 and Section 5.7).

Application Sector	Primary Stability Issue	Retention Requirement	Reported Architectures
Polyolefin packaging and films (Section 5.1)	Food-contact migration limits; formation of non-intentionally added substances (NIAS)	Near-zero migration into food simulants; regulatory compliance	Covalently bound copolymers and grafted polyolefins (Section 4.1 and Section 4.2)
Pipes, geosynthetics, infrastructure (Section 5.2)	Extraction by water and chlorinated water over 50–100-year design lives	Validated retention after aggressive water/solvent extraction	Reactive-grafted phenols (PEXa); linear oligomeric phenols
Electrical and electronic applications (Section 5.3)	Long-term thermo-oxidation at elevated temperature; dielectric integrity	Low volatility; no migration to electrical interfaces	Copolymer-bound phenols (e.g., capacitor-grade PP-HP)
Elastomers (Section 5.4)	High chain mobility; dynamic strain; contact with extractive fluids	Backbone-bound retention under flexing and fluid contact	Backbone-grafted phenols (Section 4.1); star-shaped thiol–ene oligomers (Section 4.3.2); phenol-functionalized carriers (Section 4.4)
Engineering plastics (Section 5.5)	High processing and service temperatures; polar media (ethanol, glycol)	Covalent anchoring resistant to polar-solvent extraction	POSS-bound and transamidation-bound phenols for PA6/PA66
Biomedical polymers (Section 5.6)	In vivo oxidative degradation of UHMWPE implants	Effective stabilization at hundreds of ppm; leachables below analytical and regulatory detection limits	Retention-first phenols (vitamin E benchmark); covalent phenols as candidates
Recycling and the circular economy (Section 5.7)	Cumulative stabilizer loss across multiple heat histories	Activity retained per reprocessing cycle; efficient restabilization	High-retention macromolecular phenols; hybrid silica carriers; bio-based restabilizers

Note: The architectures listed are those demonstrated in the cited literature for each sector; the mapping reflects the availability of published studies rather than direct head-to-head comparisons under sector-relevant conditions, and no ranking of architectures within or across sectors is implied.

## Data Availability

No new data were created or analyzed in this study. Data sharing is not applicable to this article.
